# Red-Light-Driven Photocatalysis with NI-BODIPY-Fullerene Systems for Organic Transformations

**DOI:** 10.1007/s10895-025-04391-y

**Published:** 2025-06-18

**Authors:** Ezel Öztürk Gündüz, Ümmügülsüm Büyükpolat, Elif Okutan

**Affiliations:** Department of Chemistry, Faculty of Science, Gebze Technical University, Gebze, Kocaeli 41400 Türkiye

**Keywords:** Borondipyrromethene, Fullerene, Napthalimide, Photocatalysis, Singlet oxygen

## Abstract

The direct use of low-energy red light for photochemical processes is attractive from a synthetic perspective but challenging to implement in practice. BODIPY-based photosensitizers (PSs) show considerable promise in this area due to their tunable photophysical properties. This study aims to explore the use of mono- and bis-adducts of heavy atom-free NI-BODIPY-fullerene triads, which strongly absorb red light in the 560 to 660 nm range, in red light-irradiated photocatalytic reactions. NI-BODIPY-fullerene photosensitizers possess the ability to generate singlet oxygen (¹O₂), making them effective photosensitizers for the photocatalytic conversion of 1,5-dihydroxynaphtalene (DHN) to juglone and the photooxidation of thioanisole to methyl phenyl sulfoxide. Our photocatalysts are effective, with activities comparable to or even higher than those of readily available commercial systems, achieving 100% conversion of thioanisole to methyl phenyl sulfoxide within 2 h. This study highlights the potential of distyryl-NI-BODIPY-fullerene derivatives as promising photosensitizers/photocatalysts for applications in organic transformations.

**Supplementary Information:**

The online version contains supplementary material available at 10.1007/s10895-025-04391-y.

## Introduction

Light driven reactions are efficient and sustainable approaches to use light energy into the preparation of valuable chemicals in mild conditions [1, 2]. In these photocatalytic reactions reactive oxygen species are involved in the breaking of bonds and adding oxygen atoms to the molecules [3]. The role of particularly singlet oxygen in photooxidation reactions has been utilized in several reaction concepts [4]. Since photocatalytic reactions generally are realized under aerobic conditions, the reactive oxygen species (generally superoxide anion radical, hydrogen peroxide, singlet oxygen, hydroxyl radical) assist in the process [5–7]. Numerous efforts have been devoted to the effective production of singlet oxygen since it has an advantageous lifetime for critical applications [8]. A major part of the ^1^O_2_ synthesis is reported from the light irradiation of heavy atoms modified organic dye [9], transition metal complexes [10], noble metal nanoparticles [11] or an organic dye bearing fullerene-C_60_ as spin converter [12]. Although functionalization with I, Br and/or Se as heavy atom is known to promote intersystem crossing (ISC) of chromophores, facilitating the sensitization of triplet molecular oxygen (^3^O_2_) into singlet oxygen, there are concerns regarding selectivity, photobleaching, self-degradation and susceptibility in the photooxidation applications [2, 13]. The ecological and economic concerns associated with the use of metal complexes and/or nanoparticles have reduced the demand for metal-based photosensitizers in photochemical applications.

BODIPY based systems are attractive triplet photosensitizers because of their easy modification and tunable photophysical/ photochemical properties [14, 15]. Heavy atom free triplet photosensitizers like BODIPY-fullerene dyads or triads applied successfully as photocatalysts rather than metal containing materials [16–19]. The introduction of BODIPY unit significantly enhances the solubility of the fullerene-C_60_ and photophysical properties making these systems more efficient over the popular conventional photocatalysts [20]. Besides, visible light absorbing (green to near IR) BODIPY-fullerene photocatalysts enable photocatalytic reactions under mild condition while enclosing expectations from an ideal photocatalyst/photosensitizers such as strong light absorption, minimal side reaction, high quantum yield and triplet energy with long-lived triplet excited state [21].

**Scheme 1:**
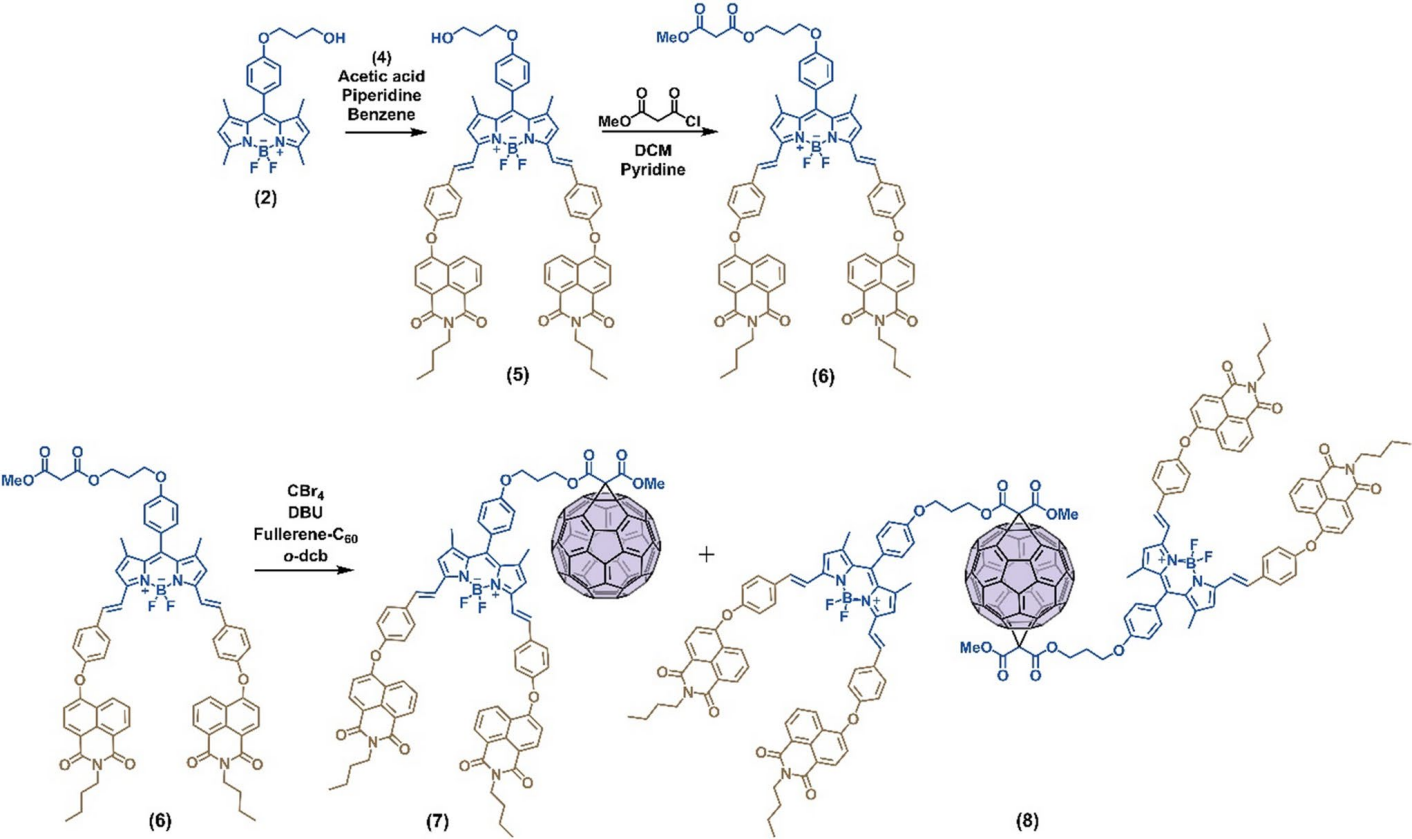
Reaction scheme to obtain NI-BODIPY-fullerene systems

In this perspective, we designed and synthesized mono- and bis adducts of distyryl-NI-BODIPY-fullerene systems (**7** and **8**) considering the important approaches such as the use of chromophores with a high molar absorption coefficient, lack of side reaction (photostability in dark conditions), induced efficient intersystem crossing, followed by the number of BODIPY units modified on fullerene in which the photophysical as well as photochemical properties are discussed and compared to correlate structure and properties. The BODIPY units are introduced to fullerene-C_60_ through modification based on the Bingel-Hirsch cyclopropanation reaction between malonyl unit on BODIPY via in situ generation of reactive monohalomalonate intermediate and [6] double bonds of the C_60_ skeleton. Here, characterizations of the BODIPYs and BODIPY-fullerenes were performed by mass spectrometry, ^1^H and ^13^C NMR spectroscopies. The photophysical properties were investigated with UV-vis absorption and 2D fluorescence emission studies. Upon light irradiation, the generation of ^1^O_2_ occurred and displayed photocatalytic oxidation in organic reactions, suggesting the synergetic effect of oxygen vacancies and light energy. The photocatalytic efficiency and stability of the catalysts were evaluated and reported during the photooxidation of DHN to juglone and thioanisole to methylphenyl sulfoxide detail. This work may serve as a foundation for further design of heavy atom free photocatalysts, aiming to achieve high efficiency and selectivity in photocatalytic organic synthesis (Scheme 1).

## General Experimental

Reagents and solvents were purchased from Sigma-Aldrich, Merck, Acros Organics, TCI or Alfa Aeser, and were used without purification unless stated otherwise.

Analytical thin layer chromatography (TLC) to follow the reactions was performed on silica gel plates (Merck, Kieselgel 60 Å, 0.25 mm thickness) with F254 indicator. Column chromatography for purification was made with silica gel (Merck, Kieselgel 60 Å, 230–400 mesh). Mass spectra were obtained in linear modes on a Bruker Daltonics Microflex mass spectrometer equipped with a nitrogen UV-laser at 337 nm. The ^1^H and ^13^C NMR spectra were acquired in deuterated solutions (CDCl_3_) with a Bruker Avance Neo 500 (500 MHz). All coupling constants (*J*) are reported in Hertz. NMR spectra signals are described as singlet (s), doublets (d), triplets (t), quintets (qu), sextets (sx) or multiplets (m). Bruker Avance Neo 400 MHz was used to obtain the ^1^H NMR spectra of the photooxidation of thioanisole. Absorption spectra of the compounds were recorded with a Shimadzu 2101 UV spectrophotometer in the UV-Vis region while indirect singlet oxygen generation measurements and photooxidation of DHN were obtained with Agilent Cary 60 UV-Vis spectrophotometer. Fluorescence emission spectra were obtained with a Varian Eclipse spectrofluorometer (1 cm path-length cuvette, RT) and the fluorescence lifetime measurements were performed by using a Horiba Jobin-Yvon-SPEX Fluorolog 3-2iHR instrument at excitation wavelengths with time-correlated single photon counting (TCSPC) module for signal acquisition.

### Synthesis

Compounds (**1**–**4**) were synthesized according to the literature (Scheme S1, ESI) [9, 22, 23].

#### Synthesis of Compound 5

In a three-necked 100 mL round-bottom flask, BODIPY derivative (**2**) (100.0 mg, 0.25 mmol) and compound **4** (282.2 mg, 0.75 mmol) were dissolved in 40 mL of benzene. Piperidine (0.30 mL) and glacial acetic acid (0.30 mL) were added. The solution was refluxed using a Dean − Stark apparatus. The reaction mixture was heated at 95 °C under reflux using a Dean–Stark apparatus to continuously remove solvent. After concentrating the solution, the reaction progress was monitored by TLC until the starting BODIPY (**2**) was fully consumed and a dark blue-colored compound became the major product. The reaction mixture was extracted three times with 200 mL of a dichloromethane–water mixture and the organic layer was dried over anhydrous Na_2_SO_4_ and concentrated on a rotary evaporator until the solvent was removed. Compound **5** was isolated by column chromatography on silica gel (230 − 400 mesh) using DCM as an eluent (65.0 mg, 23%).

*Spectral data of****5***: MS (MALDI-TOF) (DIT) (m/z): Calc. for C_68_H_59_BF_2_N_4_O_8_: 1109.05; found: 1108.764 [M]^+^, 1089.898 [M-F]^+^ (Fig. S13). ^1^H NMR (500 MHz, CDCl_3_, 293 K, δ ppm): 8.67 (d, *J* = 7.18 Hz, 2 H, Ar-CH), 8.65 (d, *J* = 7.06 Hz, 2 H, Ar-CH), 8.47 (d, *J* = 8.25 Hz, 2 H, Ar-CH), 7.78 (t, *J* = 7.83 Hz, 2 H, Ar-CH), 7.72 (d, *J* = 16.26 Hz, 2 H, *trans*-C = H), 7.72 (d, *J* = 8.16 Hz, 4 H, Ar-CH), 7.27 (d, *J* = 16.57 Hz, 2 H, *trans*-C = H), 7.23 (d, *J* = 8.56 Hz, 2 H, Ar-CH), 7.19 (d, *J* = 8.64 Hz, 4 H, Ar-CH), 7.05 (d, *J* = 8.53 Hz, 2 H, Ar-CH), 6.99 ( d, *J* = 8.25, 2 H, Ar-CH), 6.67 (s, 2 H, pyrrole-CH), 4.21 (t, *J* = 5.99 Hz, 2 H, -OCH_2_), 4.17 (t, *J* = 7.55 Hz, 4 H, -NCH_2_), 3.93 (t, *J* = 5.88 Hz, 2 H, -CH_2_O), 2.12 (qu, *J* = 5.94 Hz, 2 H, -CH_2_-), 1.71 (qu, *J* = 7.60 Hz, 4 H, -CH_2_-), 1.53 (s, 6 H, -CH_3_), 1.44 (sx, *J* = 7.48 Hz, 4 H, -CH_2_-), 0.97 (t, *J* = 7.37 Hz, 6 H, -CH_3_) (Fig. S14). ^13^C NMR (126 MHz, CDCl_3_, 293 K, δ ppm): 164.37, 163.74, 159.53, 159.34, 155.48, 152.26, 142.52, 139.50, 134.66, 134.07, 133.96, 132.68, 131.93, 129.74, 129.62, 129.43, 128.48, 127.17, 126.63, 124.04, 122.75, 120.87, 119.61, 117.82, 117.09, 115.11, 111.22, 65.68, 60.41, 60.28, 53.43, 40.20, 32.03, 30.25, 20.40, 14.94, 13.86 (Fig. S15).

#### Synthesis of Compound 6

Compound **5** (80 mg, 0.07 mmol) were dissolved in 20 mL of DCM. Pyridine (6.49 mg, 0.08 mmol) was added under Ar atmosphere and stirred for 15 min. The mixture was cooled on an ice bath and monomethyl malonyl chloride (9.83 mg, 0.07 mmol) was added. The mixture was stirred for 5 h. The reaction mixture was extracted with dichloromethane: water (200 mL, 3 times), and the organic layer was dried over anhydrous Na_2_SO_4_ and concentrated on a rotary evaporator until the solvent was removed. Compound **6** was isolated from column chromatography on silica gel (230–400 mesh) by using DCM: MeOH (200:1; v: v) as an eluent (48.0 mg, 56%).

*Spectral data of****6***: MS (MALDI-TOF) (NOM) (m/z): Calc. for C_72_H_63_BF_2_N_4_O_11_: 1209.12; found: 1209.208 [M]^+^ (Fig. S16). ^1^H NMR (500 MHz, CDCl_3_, 293 K, δ ppm): 8.66 (d, *J* = 7.28 Hz, 2 H, Ar-CH), 8.65 (d, *J* = 7.19 Hz, 2 H, Ar-CH), 8.46 (d, *J* = 8.25 Hz, 2 H, Ar-CH), 7.77 (t, *J* = 7.83 Hz, 2 H, Ar-CH), 7.71 (d, *J* = 16.25 Hz, 2 H, *trans*-C = H), 7.71 (d, *J* = 8.69 Hz, 4 H, Ar-CH), 7.28 (d, *J* = 16.21 Hz, 2 H, *trans*-C = H), 7.23 (d, *J* = 8.53 Hz, 2 H, Ar-CH), 7.19 (d, *J* = 8.62 Hz, 4 H, Ar-CH), 7.04 (d, *J* = 8.58 Hz, 2 H, Ar-CH), 6.99 ( d, *J* = 8.25, 2 H, Ar-CH), 6.67 (s, 2 H, pyrrole-CH), 4.42 (t, *J* = 6.30 Hz, 2 H, -OCH_2_), 4.17 (t, *J* = 7.55 Hz, 4 H, -NCH_2_), 4.13 (t, *J* = 6.12 Hz, 2 H, -CH_2_O), 3.76 (s, 3 H, -OCH_3_), 3.43 (s, 2 H, -CH_2_-), 2.21 (qu, *J* = 6.19 Hz, 2 H, -CH_2_-), 1.71 (qu, *J* = 7.60 Hz, 4 H, -CH_2_-), 1.53 (s, 6 H, -CH_3_), 1.44 (sx, *J* = 7.48 Hz, 4 H, -CH_2_-), 0.97 (t, *J* = 7.37 Hz, 6 H, -CH_3_) (Fig. S17). ^13^C NMR (126 MHz, CDCl_3_, 293 K, δ ppm): 166.97, 166.48, 164.35, 163.72, 159.44, 159.33, 155.48, 152.26, 142.50, 139.45, 134.68, 134.06, 133.95, 132.67, 131.91, 129.72, 129.63, 129.43, 128.47, 127.22, 126.62, 124.02, 122.74, 120.87, 119.60, 117.83, 117.08, 115.09, 111.20, 64.31, 62.39, 53.44, 52.58, 41.33, 40.19, 30.25, 28.54, 20.40, 14.94, 13.86 (Fig. S18).

#### Synthesis of Compound 7 and 8

BODIPY derivative (**6**) (30.0 mg, 0.02 mmol) was dissolved in 20 mL *o*-dcb in a two-necked round-bottom flask. Fullerene-C_60_ (27 mg, 0.04 mmol) and CBr_4_ (8.30 mg, 0.02 mmol) were added to this solution. The reaction mixture was stirred for 30 min. at room temperature. DBU (7.60 mg, 0.05 mmol) in 10 mL *o*-dcb was added to the reaction mixture drop by drop and reaction mixture was stirred for another 5 h. Compounds **7** and **8** were isolated by column chromatography on silica gel (230–400 mesh) by using DCM: MeOH (200:1; v: v) as an eluent (**7**: 15.0 mg, 31%, **8**: 8.0 mg, 10%).

*Spectral data of****7***: MS (MALDI-TOF) (DIT) (m/z): Calc. for C_132_H_61_BF_2_N_4_O_11_: 1927.76; found: 1927.65 [M]^+^ (Fig. S19). ^1^H NMR (500 MHz, CDCl_3_, 293 K, δ ppm): 8.68 (d, *J* = 6.1 Hz, 2 H, Ar-CH), 8.67 (d, *J* = 6.9 Hz, 2 H, Ar-CH), 8.48 (d, *J* = 8.2 Hz, 2 H, Ar-CH), 7.78 (t, *J* = 7.9 Hz, 2 H, Ar-CH), 7.73–7.71 (m, 2 H, *trans*-C = H + 4 H, Ar-CH), 7.28 (d, *J* = 17.4, 2 H, *trans*-C = H), 7.20 (d, *J* = 7.5 Hz, 4 H, Ar-CH), 7.07 (d, *J* = 8.2 Hz, 2 H, Ar-CH), 7.00 (d, *J* = 8.2 Hz, 2 H, Ar-CH), 6.67 (s, 2 H, pyrrole-CH), 4.77 (t, *J* = 5.1 Hz, 2 H, -OCH_2_), 4.23 (t, *J* = 5.8 Hz, 2 H, -OCH_2_), 4.18 (t, *J* = 7.3 Hz, 4 H, -NCH_2_), 4.09 (s, 3 H, -OCH_3_), 2.45–2.42 (m, 2 H, -CH_2_-), 1.74–1.68 (m, 4 H, -CH_2_-), 1.53 (s, 6 H, -CH_3_), 1.48–1.42 (m, 4 H, -CH_2_-), 0.97 (t, *J* = 7.5 Hz, 6 H, -NCH_3_) (Fig. S20). ^13^C NMR (126 MHz, CDCl_3_, 293 K, δ ppm): 164.33, 164.07, 163.70, 163.57, 159.36, 159.29, 159.27, 155.48, 152.28, 145.31, 145.24, 145.20, 145.07, 145.04, 144.96, 144.93, 144.72, 144.70, 144.66, 144.61, 143.90, 143.86, 143.13, 143.07, 143.05, 142.94, 142.38, 142.20, 142.17, 141.93, 141.84, 141.00, 138.78, 134.69, 134.02, 133.89, 132.64, 131.90, 129.72, 129.41, 128.45, 126.60, 124.02, 122.74, 120.84, 120.70, 117.09, 40.18, 30.25, 20.39, 14.97, 13.85 (Fig. S21).

*Spectral data of****8***: MS (MALDI-TOF) (CHCA) (m/z): Calc. for C_204_H_122_B_2_F_4_N_8_O_22_: 3134.87; found: 3134.16 [M]^+^ (Fig. S22). ^1^H NMR (500 MHz, CDCl_3_, 293 K, δ ppm):: 8.66 (d, *J* = 5.1 Hz, 4 H, Ar-CH), 8.65 (d, *J* = 6.6 Hz, 4 H, Ar-CH), 8.46 (d, *J* = 8.2 Hz, 4 H, Ar-CH), 7.77 (t, *J* = 7.8 Hz, 4 H, Ar-CH), 7.71–7.69 (m, 4 H, *trans*-C = H + 8 H, Ar-CH), 7.26 (d, *J* = 15.48 Hz, 4 H, *trans*-C = H), 7.18 (d, *J* = 7.8 Hz, 8 H, Ar-CH), 7.05–6.97 (m, 8 H, Ar-CH), 6.66 (s, 4 H, pyrrole-CH), 4.73–4.63 (m, 4 H, -OCH_2_), 4.22–4.09 (m,18 H, -OCH_2_+-NCH_2_ +-OCH_3_), 1.70 (qu, *J* = 7.5 Hz, 12 H, -CH_2_-), 1.52 (s, 12 H, -CH_3_), 1.44 (sx, *J* = 7.4 Hz, 8 H, -CH_2_-), 0.96 (t, *J* = 7.3 Hz, 12 H, -NCH_3_) (Fig. 23). ^13^C NMR (126 MHz, CDCl_3_, 293 K, δ ppm): 164.48, 163.85, 159.44, 155.63, 152.42, 146.69, 146.58, 145.56, 144.90, 143.96, 143.66, 142.57, 142.12, 139.44, 134.90, 134.16, 134.05, 132.79, 132.06, 129.86, 129.56, 128.60, 126.76, 124.15, 122.88, 121.02, 119.68, 118.00, 117.22, 115.24, 111.32, 53.57, 40.34, 32.07, 30.39, 29.84, 20.54, 15.13, 14.00 (Fig. S24).

### Photophysical Properties

Fluorescence quantum yield values of NI-BODIPY-C_60_ systems (**7** and **8**) were determined in the DCM by comparitive method. Cresyl violet (Φ_F_ = 0.56 in ethanol) was studied as reference and fluorescence quantum yield values (ΦF_sample_) were determined by using Eq. (1) [24].

1 $$\:{{\Phi\:}{F}_{sample}={\Phi\:}F}_{ref}\times\:\frac{{F}_{sample\:}\times\:\:{A}_{ref}\:\times\:\:{{\eta\:}_{sample}}^{2}}{{F}_{ref}\:\times\:{\:A}_{sample}\:\times\:\:{\eta\:}_{ref}^{2}}$$

F: area under the fluorescence emission curve.

A: absorbance values at the excitation wavelength.

ƞ: refractive index of the solvents.

Lifetime values of both photosensitizers (**7** and **8**) were calculated by using 2-exponentials and the acceptability of the fits was judged by χ2 criteria. Hence, mean (average) lifetime values (τ_a_) determined according to the Eq. (2) where τ_i_ is the decay time constant and α_i_ is the pre-exponential factor [25].

2 $$\:{\tau\:}_{a}=\:\frac{\sum\:{\alpha\:}_{i}\:{{\tau\:}_{i}}^{2}}{\sum\:{\alpha\:}_{i}{\tau\:}_{i}}$$

### Determination of Singlet Oxygen Quantum Yields

Indirect method was applied to determine the singlet oxygen quantum yields of **7** and **8** in DCM. In this method, DPBF was used as trap molecule. Photosensitizer and DPBF containing solution was irradiated with red LED (630 nm, 4.0 mW/cm^2^) from a 10.0 cm distance and absorbance spectra of the solution was recorded at regular intervals. MB was used as a reference triplet photosensitizer with 0.57 singlet oxygen quantum yield in DCM [19] and singlet oxygen quantum yields were calculated according to Eq. 3. In the equation given below, k represents the slope of the graph depicting the decrease in absorbance value of DPBF over time. F is calculated by the given formula which is F = 1–10^− O.D^. (O.D. is the absorbance value at irradiation wavelength) and PF is the light intensity of the irradiation source (energy flux, mW/cm^2^).

3 $$\begin{aligned}\:{\Phi _\Delta }\left( {{\text{sample}}} \right) = \Phi {\:_{\Delta \:}}\left( {{\text{ref}}} \right) \times \:\:\frac{{{\text{k}}\left( {{\text{sample}}} \right)}}{{{\text{k}}\left( {{\text{ref}}} \right)}} \hfill\times \:\frac{{{\text{F}}\:\left( {{\text{ref}}} \right)}}{{{\text{F}}\:\left( {{\text{sample}}} \right)}} \times \:\frac{{{\text{PF}}\:\left( {{\text{ref}}} \right)}}{{{\text{PF}}\:\left( {{\text{sample}}} \right)}} \hfill \\ \end{aligned} $$

### Singlet Oxygen Quenching Experiment

Singlet oxygen quenching experiments were carried out in the presence of sodium azide by using Horiba Jobin-Yvon Fluoremeter with Hamamatsu NIR PMT 5509. Increasing concentration of sodium azide solution in water were added cascade into the solutions of compounds **7** and **8** in THF and the decrease in the singlet oxygen phosphorescence at 1270 nm were recorded.

### Photooxidation of DHN

Photooxidation of DHN experiments were carried out in DCM: MeOH (9:1; v: v; 10 mL) solvent mixture. Three different solutions were prepared in a vial, consisting of **7**, **8**, and **MB** (5 mol % relative to DHN) respectively as photosensitizers, along with DHN (2 × 10⁻⁴ M). The solution containing DHN and the photosensitizer was irradiated by 630 nm red LED for 90 min and spectral changes were recorded every 10 min using a UV-Vis absorbance spectrophotometer. The photooxidation of DHN was monitored by the decrease in absorbance at 301 nm. Moreover, the formation of 5-hydroxy-1,4-naphthoquinone (juglone) from DHN was determined by the increase in absorbance at 427 nm. Yield of juglone was calculated according to the Eq. 4 given below where c_juglone_ is the concentration of juglone and c_DHN_ is the initial concentration of DHN. Concentration of these two compounds were calculated by using their molar absorptivity coefficients (Ɛ_DHN_= 7664 M^-1^cm^-1^; Ɛ_juglone_=3811 M^-1^cm^-1^).

4 $$\:\text{Y}\text{i}\text{e}\text{l}\text{d}\:\text{o}\text{f}\:\text{J}\text{u}\text{g}\text{l}\text{o}\text{n}\text{e}\:\text{\%}=\:\frac{{\text{C}}_{\text{j}\text{u}\text{g}\text{l}\text{o}\text{n}\text{e}}}{{\text{C}}_{\text{D}\text{H}\text{N}}}\times\:100$$

### Photocatalytic Oxidation of Thioanisole

Methanol: DCM (55:45; v: v, 1 mL), degassed with oxygen for 30 min, solution containing NI-BODIPY-C_60_ catalysts (6.67 × 10^− 4^ mmol, 1 equiv.) and thioanisole (0.1 mmol, 150 equiv.) were added to a 3 mL vial equipped with a magnetic stir bar. The reaction mixture was stirred at room temperature at a distance of 30 cm from a 12 W red LED (660 nm, 28.8 mW/cm^2^). Aliquots were taken after 2 hours of irradiation. ^1^H NMR spectrum was taken of the reaction mixture. Conversion yields were calculated by the ratio of integrated intensity between the ^1^H NMR peaks of the thioanisole and phenyl methyl sulfoxide.

## Results and Discussion

### Synthesis and Structural Characterization

Compounds **1**–**4** were obtained according to the previously reported literature, characterized and used in subsequent steps [9, 22, 23]. In order to tune the photophysical properties of BODIPY core and impart the NI unit, Knoevenagel condensation in benzene was applied and distyryl NI-BODIPY (**5**) was synthesized. Characteristic doublet peaks corresponding to the *trans*-C = H protons were observed in the ^1^H NMR spectrum with a ~ 16 Hz coupling constant in the aromatic region (Fig. S14, ESI). Following this step, the reaction between **5** and commercial monomethyl malonyl chloride resulted in compound **6**, which was then used to prepare **7** and **8** in the final step of the synthesis. The presence of -CH_2_- in the carbonyl units and -OCH_3_ groups distinguishes the ^1^H NMR spectrum of compound **6** from **5** (Fig. S17, ESI). The Bingel reaction, a [2 + 1] cycloaddition mechanism commonly used to prepare multi-adduct fullerene-C_60_ derivatives, was employed to synthesize mono- and bis-adduct NI-BODIPY-fullerene triads **7** and **8**. The bis-adduct NI-BODIPY-C_60_ derivative was prepared through a non-regioselective one-pot synthesis, where the final product theoretically includes eight different isomers: *cis*-1, *cis*-2, *cis*-3, *e*, *trans*-1, *trans*-2, *trans*-3, and/or *trans*-4. Compound **7** was obtained with a yield of 31%, while **8** had a yield of 10%. Indeed ^1^H NMR spectra of both **7** and **8** confirmed the cyclopropanation in the final structures where the singlet -CH_2_- peak at 3.43 ppm vanished (Fig. S8 and S11, ESI). Furthermore, sp^2^ hybridized C atoms of fullerene-C_60_ were distinguished from each other by functionalization; hence, peaks of sp^2^ hybridized C atoms resonated between 145 − 141 ppm in the ^13^C NMR spectra for both cases (Fig. S21 and S24, ESI). In particular, we also confirmed the multistep synthesis and functionalization through mass spectrometry analysis.

### Photophysical Properties

**Fig. 1:**
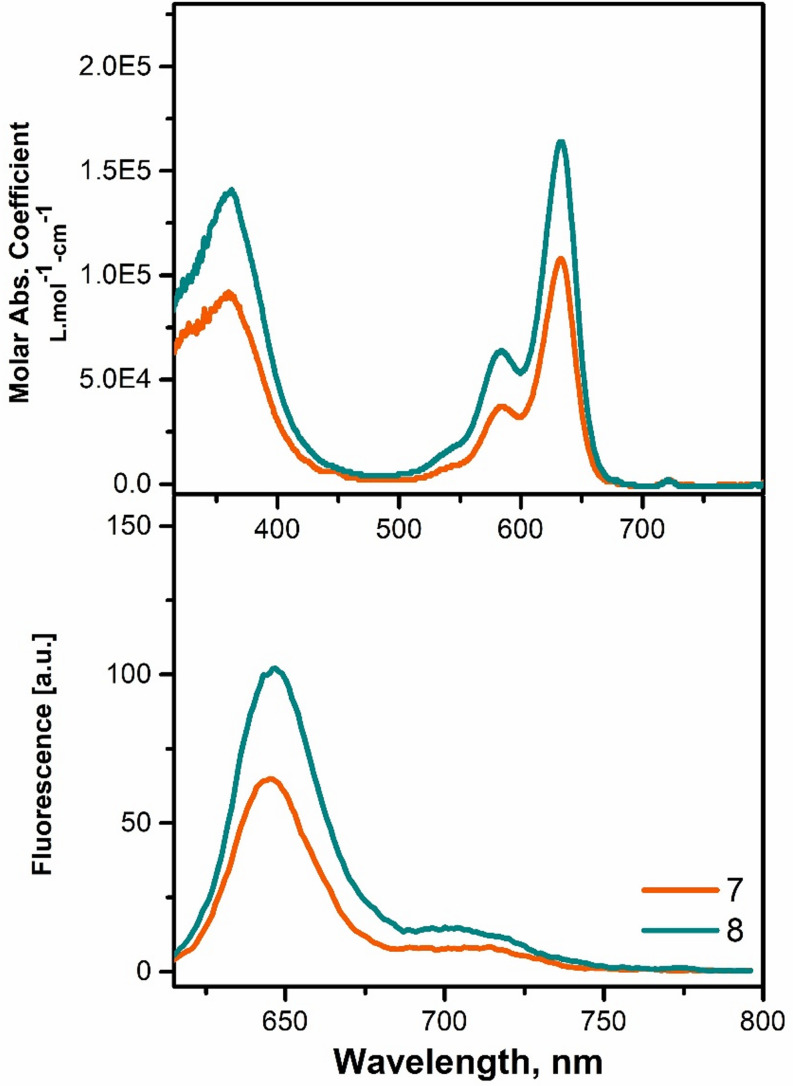
UV-vis absorption and emission spectra of triads (1µM) in DCM

The steady-state absorption and emission properties of the target molecules, NI-BODIPY-C_60_ (**7** and **8**) have been investigated in order to evaluate the overall photophysical features of the triads **7** and **8**. In addition, to inquire about the fluorescence dynamics of **7** and **8**, fluorescence decay traces were collected as functions of emissions wavelengths λ_em_. The steady-state absorption and emission spectra of the molecules **7** and **8** in various solvents and solvent mixture including DCM, DMSO, THF, MeOH, Toluene, DCM: MeOH (9:1; v: v) and DMSO: water (5:95; v: v) revealed the same trend observed in reported NI-BODIPYs and BODIPY-fullerene systems [17, 26]. The intense absorption bands, located at ~ 360 and ~ 634 nm in DCM, THF and DCM: MeOH (9:1; v: v), are undoubtedly assigned to the methanofullerene and S_0_-S_2_/ S_0_-S_1_ and vibronic (S_0_-S_1,1,_ S_0_-S_1,2_) transitions of the BODIPY respectively (Fig. 1). Small red shifts were seen in the absorption spectra in DMSO, MeOH and DMSO: water (5:95; v: v). In addition, when triads were excited at 600 nm in selected solvents, the emission band of the BODIPY moieties were found to be quenched in MeOH and DMSO: water (5:95; v: v) and gave slight emission between 640 and 650 nm in other solvents which is in analogy with the behavior obtained for the chromophore-fullerene systems [17]. A lower energy band between 700 and 750 nm can be seen for compounds **7** and **8** that may be predominantly attributed to vibronic fluorescence which may also be assigned to the emission of C_60_ unit [27]. The fluorescence quenching effect of C_60_ was observed in the fluorescence lifetime values as well (Fig. S31, ESI). Both triads displayed bi-exponential decays. Long-lived decay time with minor amplitude contribution of compound **7** (2.37 ns, 12%) and compound **8** (2.68 ns, 27%) can be attributed to the singlet excited of BODIPY (^1^BDP^*^). Shorter lifetime values with major amplitude contributions of compound **7** (0.33 ns, 88%) and compound **8** (0.44 ns, 73%) shows excited-state quenching due to the energy and/or electron transfer from ^1^BDP^*^ to C_60_ moiety in the structure [28–30]. Furthermore, the average lifetime values were calculated as 1.34 ns and 1.99 ns for compounds 7 and 8, respectively, using Eq. 2. The results were given in Table 1. These results confirmed the influence of the fullerene on the photophysical properties. Light absorption efficiencies of the compounds **7** and **8** were investigated in DCM at various concentrations. Different solutions with various concentrations were prepared and absorption spectra were recorded. Maximum absorbance values at 633 nm were plotted against concentrations and molar absorptivity (Ɛ) values of the triads were determined to be 11.26 × 10^4^ M^− 1^cm^− 1^ and 17.41 × 10^4^ M^− 1^cm^− 1^ for **7** and **8**, respectively. Both triads followed the Lambert-Beer Law, and as expected, the bis-adduct of NI-BODIPY fullerene (**8**) exhibited a higher molar absorption coefficient than the mono-adduct (**7**) where increased absorbance may lead to better light harvesting and improved photocatalytic performance (Fig. S27 and S30, ESI).

**Table 1:** Photophysical parameters of compounds **7** and **8**

Compounds	λ_abs_ (nm)	λ_ems_ (nm)	Δ_stokes_ (cm^− 1^)	^a^Ɛ	^b^Φ_F_	^c^χ²	^d^τ_F_ (ns)	
**7**	360, 633	645	294	11.26	0.04	0.89	0.33 2.37	88% 12%
**8**	360, 633	647	342	17.41	0.04	1.12	0.44 2.68	73% 27%

^**a**^Molar extinction coefficients (x10^4^ M^− 1^cm^− 1^) in DCM, ^**b**^Fluorescence quantum yield in DCM, ^c^CHISQ ^**d**^Fluorescence lifetime values with their amplitude contributions in DCM

### Photochemical and Photocatalytic Properties

**Fig. 2:**
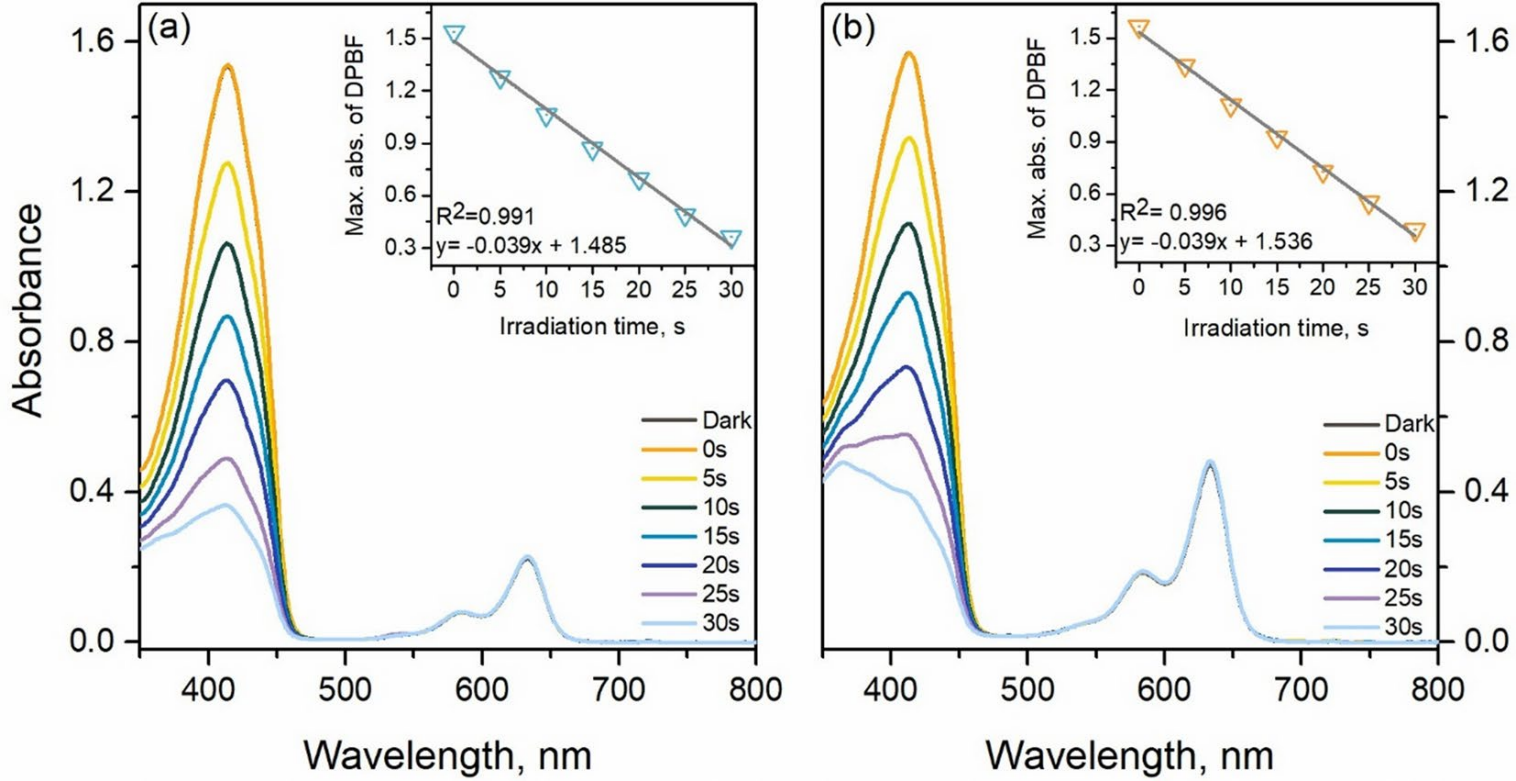
Gradual absorbance decreases of DPBF in the presence of (**a**) compound **7** and (**b**) compound **8** (2 µM)

Catalysis plays a critical part in transformation of feedstocks into valuable products such as drugs, polymers or biomolecules in chemical industry and catalysts are being used over 90% of chemical production processes worldwide. A new trend is carrying out these chemical transformations “green” for sustainable Earth. Developing catalysts to achieve improved product yields and better selectivity (chemo-, regio-, or stereo-) has been at the core of these efforts [31]. In this regard, photocatalysis has been extensively studied over the past decade and offers an eco-friendly approach to transformations in organic reactions [32]. UV and blue light are mostly utilized as light sources and photocatalysts used so far have several limitations. While some transition-metal-based complexes are limited to the UV region, greatly reducing their efficiency, others, despite being extensively studied for their photophysical and electrochemical properties, are relatively expensive [33, 34]. Several organic dyes such as methylene blue, eosin Y, Rhodamine 6G and various acridinium salts have been used successfully as photocatalysts in the light-mediated reactions [35, 36]. Organic photocatalysts generally follow two mechanisms: the excited photocatalyst can initiate oxidations via electron transfer producing a photocatalyst and/or a cation radical or photocatalysts with high triplet yields generate singlet oxygen (^1^O_2_), to form superoxide (O_2_^•–^) and a cation radical. The photophysical processes in BODIPY–C₆₀ systems have been described in detail [37–39]. As previously discussed for BODIPY–fullerene dyad systems, photoexcitation leads to the formation of the singlet excited state, ¹BODIPY–C₆₀, which can then undergo intersystem crossing (ISC) to populate the triplet excited state, ³BODIPY–C₆₀, or proceed via charge separation to generate a radical pair, ³BODIPY⁺–C₆₀⁻. This radical pair can subsequently recombine to yield neutral triplet states of either C₆₀ or BODIPY. Alternatively, the radical pair may undergo hole separation, followed by charge recombination to the ground state, particularly in polar solvents. Thus, herein we have first acquired the values of singlet oxygen quantum yields, Φ_Δ_, via indirect method by using DPBF as trap molecule where MB (Fig. S33) was used as reference to calculate Φ_Δ_ values. Quantum yields for singlet oxygen generation in dichloromethane were 0.45 and 0.28 for **7** and **8** respectively (Fig. 2; Table 2).

Sodium azide is a very well-known physical quencher of singlet oxygen, and it is frequently used to determine singlet oxygen generation [40, 41]. To verify the production of singlet oxygen, NaN_3_ solution was added to the solutions of compound **7** and **8**, respectively and singlet oxygen phosphorescence at 1270 nm were recorded. The decrease in phosphorescence intensity with increasing NaN_3_ concentration clearly indicates the quenching of singlet oxygen confirming the generation of singlet oxygen in these systems (ESI, Fig. S36).

**Table 2:** Photochemical properties of compounds **7** and **8**

Compound	^a^Φ_Δ_	^b^k_obs_	^c^ν_i_	% Yield of Juglone
**7**	0.45	0.004	8.0	63
**8**	0.28	0.002	4.0	47

^**a**^Singlet oxygen quantum, yield ^**b**^Pseudo-first-order rate constant (10^–3^ min ^–1^), ln(A_t_/A_0_) =–k_obs_ t. ^**c**^Initial consumption rate (10^–7^ M min ^–1^) of DHN, ν_i_ = k_obs_ [DHN]

**Scheme 2:**
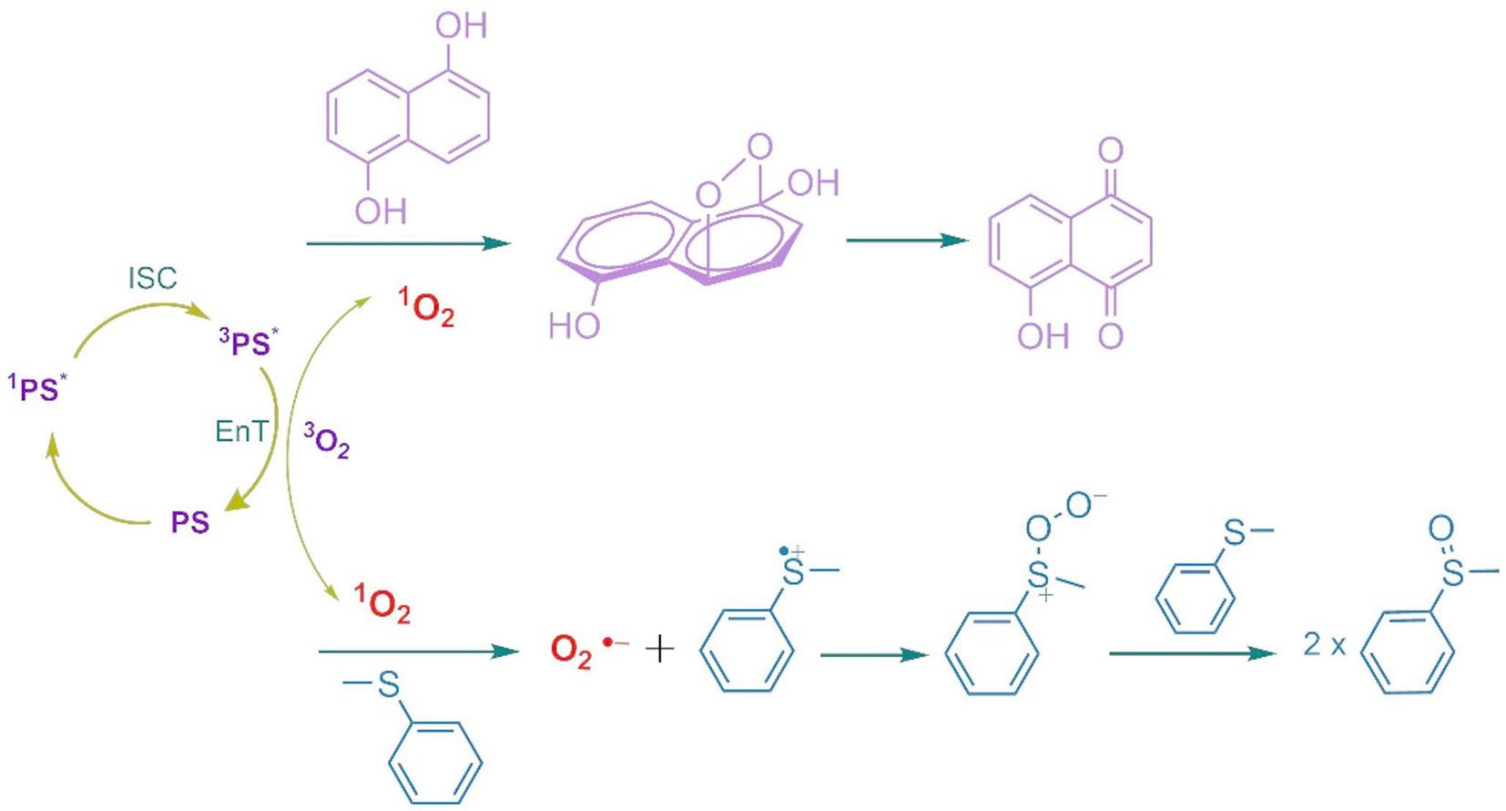
Photocatalyzed oxidation mechanisms of DHN and thioanisole

**Fig. 3:**
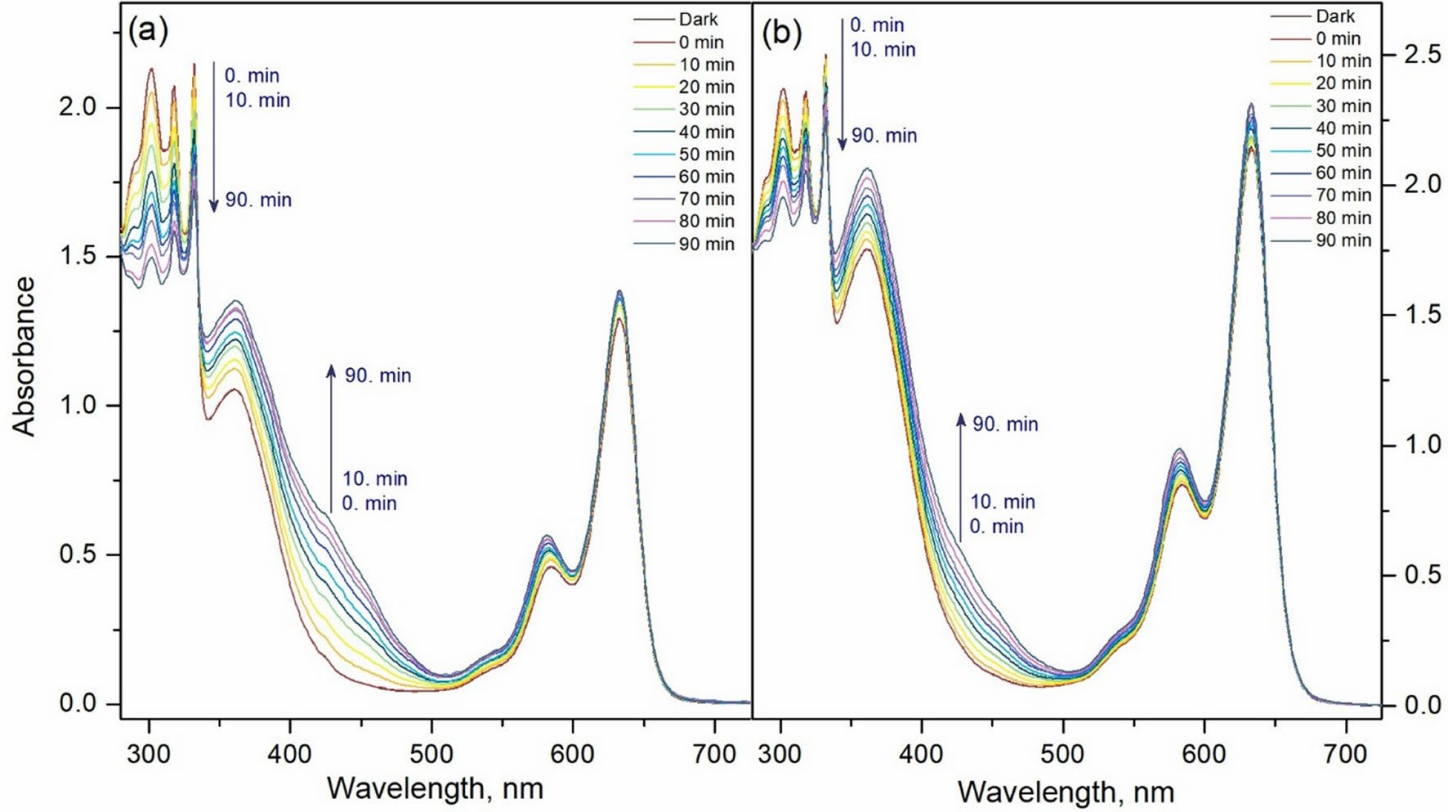
A representative set of UV-vis spectra of DHN in DCM: MeOH (9:1; v: v) recorded during in situ illumination of (**a**) **7** and (**b**) **8** with a red LED

**Fig. 4:**
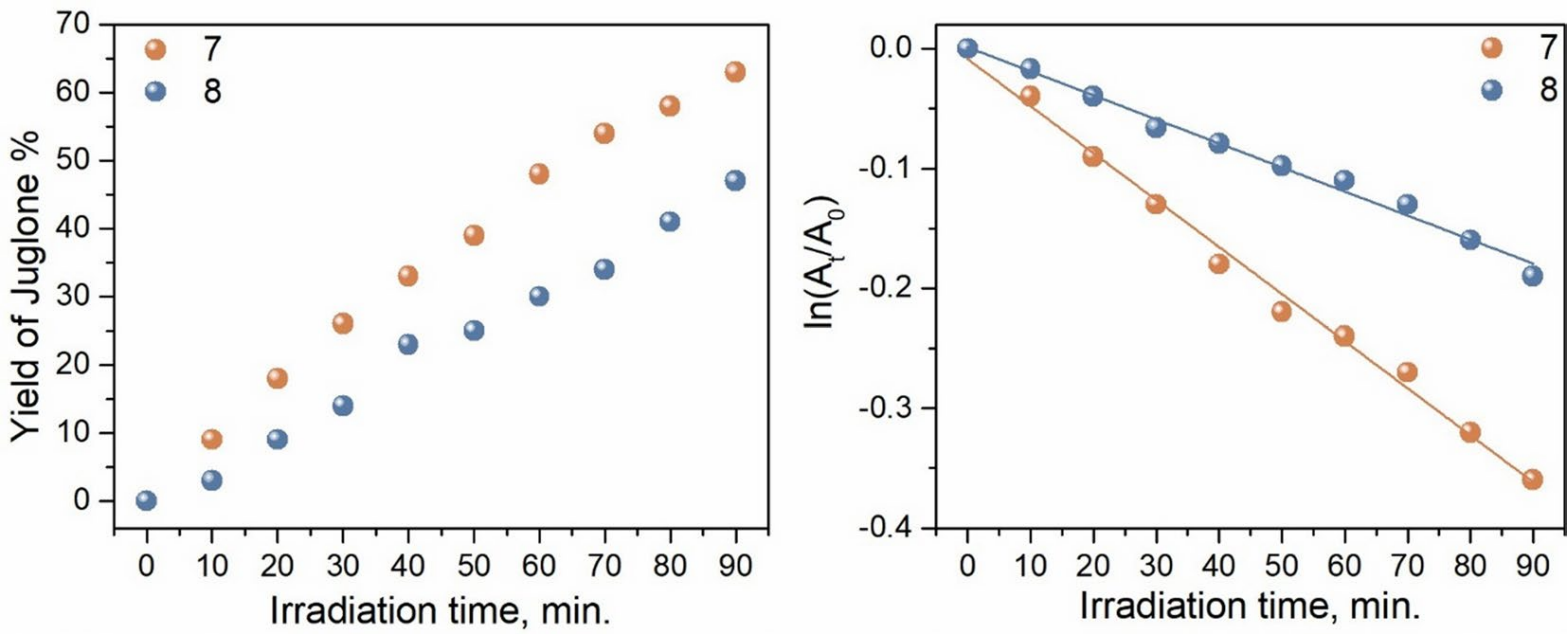
Plots of % yield of juglone and ln(A_t_/A_0_) vs. irradiation time (t) for the photooxidation of DHN

Due to their effective light absorption in the red-light range and ability to generate singlet oxygen, a study was conducted on the photocatalytic activity of compounds **7** and **8** under light irradiation, demonstrating their potential in the transformation of 1,5-dihydroxynaphthalene to juglone as an example of fine-chemical synthesis (Scheme 2) [42, 43]. Juglone, 5-hydroxy-1,4-naphthoquinone, as an antimicrobial agent, naturally occurs in plants, particularly in black walnut [44]. This is achieved by selectively applying photoexcitation in situ to compounds **7** or **8** (5% mole) in DHN solution (DCM: MeOH; 9:1, v: v) to generate singlet oxygen (¹O₂), resulting in a noticeable change in the UV–vis absorption spectra. The oxidation of DHN to juglone served as a proof of concept for directly measuring the species formed in the presence of singlet oxygen. The oxidation process of DHN can be easily monitored and quantified by the decrease in DHN absorbance peaks, along with the appearance of juglone absorption in the UV–vis spectra (Fig. 3). The photooxidation rates using **7** and **8** as triplet PSs were compared by plotting the ln(A_t_/A_0_)/ t, where A_t_ is the absorption, and the t is the irradiation time (Fig. 4). Distinct differences could be observed between the slopes (k_obs_) of the photooxidation reactions when using different systems. Naturally the slope of **8** was much smaller than that of **7** (Table 2). Significantly, the photooxidation capacity of compound **7** was much stronger than that of the bis adduct **8** when used as a photosensitizer (PS) and photocatalyst. This suggests that synthesizing multiple adducts to enhance molar absorption coefficients and facilitate triplet excited state formation may lead to unforeseen results in the photocatalytic application for organic reactions. The initial consumption rate of DHN (ν_i_) and the yield of juglone were also determined. For compound **7**, the initial consumption rate was found to be ν_i_ = 8.0 × 10^− 7^ M min^− 1^, with a juglone yield of 63% after 90 min. In contrast, for another **8**, the reaction proceeded more slowly (ν_i_ = 4.0 × 10^− 7^ M min^− 1^), resulting in a lower juglone yield of 47%. These findings align with the light-harvesting and photophysical properties, as well as the singlet oxygen quantum yields. Moreover, the photocatalytic performance, with a moderate yield of juglone, was comparable to previously reported photocatalysts, although the irradiation time increased with respect to both the photocatalyst’s molar amount and/or the energy flux of the light source [44, 45].

**Fig. 5:**
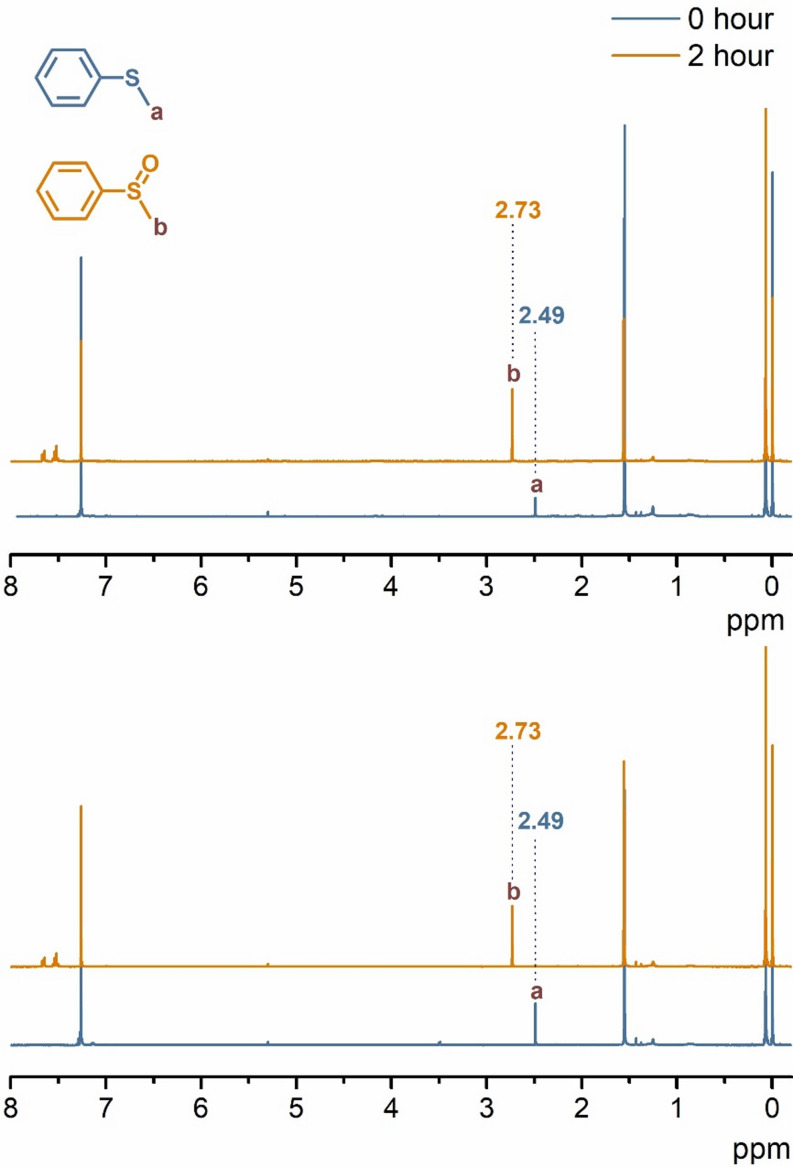
Spectral changes of thioanisole in ^1^H NMR in the presence compound **7** (top) and compound **8** (bottom) in CDCl_3_

Sulfoxide production from oxidation of sulfides is one of the most essential process since sulfoxide derivatives are used tremendously as an intermediate in especially pharmaceutical and fragrance industry [46–48]. Hence, the photocatalytic performance of the NI-BODIPY-C_60_ triads was further investigated for the selective aerobic oxidation of thioanisole (methyl phenyl sulfide) to phenyl methyl sulfoxide (Scheme 2). 660 nm red LED was used to irradiate the thioanisole-catalyst mixture from a distance of 30 cm. In catalytic reactions, solvent has a significant part. In this study, methanol was chosen as a reaction solvent as it has been previously reported to provide high conversion yield for the photooxidation of thioanisole [46]. However, in this study methanol-dichloromethane solvent mixture was used due to the low solubility of triads in methanol. Photocatalysis of thioanisole were carried out at room temperature with solutions only containing thioanisole and BODIPY-C_60_ derivatives. As shown in Table 3, both compound **7** and **8** showed extraordinary photocatalytic activity for the aerobic oxidation of thioanisole achieving 100% conversion after only 2 h of irradiation [47]. The conversion was determined by analyzing the ^1^H NMR spectra of the reaction mixtures. It was observed that the -CH_3_ peak of thioanisole at 2.49 ppm had completely disappeared and a new peak was observed at 2.73 ppm corresponding to the -CH_3_ protons attached to the sulfoxide group (Fig. 5).

Several photocatalysts have been reported for the photooxidation of thioanisole; however, while some of them require much more energetic light sources, others achieve 100% conversion over longer reaction times [46, 49–51]. In this respect, compounds **7** and **8** demonstrated highly satisfactory photocatalytic function with 100% conversion within 2 h using a lower energy source (red LED).

**Table 3:** Photooxidation of thioanisole in the presence of compounds **7** and **8**

Catalyst	Conversion %^a^
7	100^b^
8	100^b^

^a^Determined by ^1^H NMR, ^b^Irradiation time was 2 h

## Conclusion

Modification of the BODIPYs with fullerene-C_60_ as universal spin converter induces structural and photophysical/ photochemical tune that enhance generation of reactive oxygen species. In particular, preparation of bis-adduct NI-BODIPY-fullerenes induces an increase of molar absorption coefficient of dyad which may expected to enhance singlet oxygen production. The mono-adduct NI-BODIPY-fullerene (**7**) produced singlet oxygen more efficiently than the two-absorbing-unit version (**8**) (Φ_Δ_ = 0.45 and 0.28 in DCM for **7** and **8** respectively), consistent with previous findings, as even minor functionalization can influence singlet oxygen formation, making it difficult to directly correlate molecular design with quantum yield. On the other hand, these triads were used in two model photochemical transformations at room temperature. We confirmed the production of juglone over DHN upon irradiation of triplet photosensitizers **7** and **8** through UV-Vis absorption experiments. Notably, the photocatalytic performance was slower than expected, but transforming DHN to juglone under mild conditions in the presence of photocatalysis to a 63 and 48% yield respectively in 90 min. Accordingly, the photocatalytic oxidation of sulfide to sulfoxide occurred with improved reaction rate in the presence of **7** or **8** under simultaneous red LED irradiation. ^1^H NMR experiments were able to easily identify the changes in the photocatalytic reactions where 100% yields were achieved under 2 h for both photosensitizers. Hence, this study concludes that both the design of photosensitizers with the desired properties and the nature of the photocatalytic reaction are essential for obtaining high yields and reaction rates, making them critical factors that must be carefully considered.

## Electronic Supplementary Material

Below is the link to the electronic supplementary material.

Supplementary Material 1

## Data Availability

No datasets were generated or analysed during the current study.
