# Supplementary material for: Red-Light-Driven Photocatalysis with NI-BODIPY-Fullerene Systems for Organic Transformations
Source: J Fluoresc. 2025 Jun 18;35(11):11651–62. doi: 10.1007/s10895-025-04391-y (PMC12718227; doi:10.1007/s10895-025-04391-y)
Supplement: Supplementary file 1 — Supplementary Material 1 [file 10895_2025_4391_MOESM1_ESM.docx]

**Red-Light-Driven Photocatalysis with NI-BODIPY-Fullerene Systems for Organic Transformations**

Ezel Öztürk Gündüz^a,*^, Ümmügülsüm Büyükpolat^a^, Elif Okutan^a^

^a^ Department of Chemistry, Faculty of Science, Gebze Technical University, Gebze, Kocaeli, Türkiye

**Scheme S1.** Synthesis of BODIPY and aldehyde derivatives.

**Synthesis of compound 1**

4-Hydroxybenzaldehyde (1.50 g, 12.00 mmol) was dissolved in acetonitrile (15 mL) in a 100 mL round-bottom three-necked flask equipped with a magnetic stirrer. Potassium carbonate (K₂CO₃, 2.20 g, 15.90 mmol) was added to the solution, and the mixture was stirred at room temperature for 20 minutes. A solution of 3-chloro-1-propanol (1.10 g, 12.00 mmol) in acetonitrile (5 mL) was then added dropwise via a dropping funnel. The reaction mixture was heated to 80 °C and stirred under reflux for 12 hours. Reaction progress was monitored by thin-layer chromatography (TLC). Upon completion, the mixture was filtered through cellulose filter paper (pore size < 2 µm), and the solvent was partially removed under reduced pressure using a rotary evaporator. The crude product was purified by column chromatography on silica gel (230–400 mesh) using a n-hexane:ethyl acetate (3:2) mixture as the eluent, yielding Compound **1** as a pure oily substance (1.20 g, 55%).

*Spectral data of* ***1***: MS (MALDI-TOF) (DHB) m/z Calc.:180.20; found: 180.041 (Fig S1). ^1^H NMR (500 MHz, CDCl_3_, 298 K, δ ppm) 9.86 (s, 1H, -CHO), 7.81 (d, *J* = 8.8 Hz, 2H, Ar-CH), 7.00 (d, *J*= 8.7 Hz, 2H, Ar-CH), 4.20 (t, *J*= 6.1 Hz, 2H, -OCH_2_), 3.86 (t, *J*= 6.0 Hz, 2H, -CH_2_O), 2.07 (m, 2H, -CH_2_-) (Fig S2). ^13^C NMR (126 MHz, CDCl_3_, 298 K, δ ppm) δ 190.86, 163.94, 132.01, 129.94, 114.76, 65.58, 59.66, 31.85 (Fig S3).

**Synthesis of compound 2**

Compound **1** (1.10 g, 5.93 mmol) and 2,4-dimethylpyrrole (1.20 g, 13.2 mmol) were dissolved in 150 mL of DCM and stirred 30 min and few drops of trifluoroacetic acid was added to the reaction mixture and stirred for 2-3 h. p-chloranil (0.87 g, 3.56 mmol) was dissolved in 100 mL of DCM and added to the reaction mixture drop by drop. The reaction mixture was stirred for 2 h at room temperature and triethylamine (8 mL, 57 mmol) was added to the mixture drop by drop and stirred for 30 min. BF_3_. OEt_2_ (8 mL, 66 mmol) was added to the mixture drop by drop. The reaction was stirred at RT 2-3 h and reaction mixture was extracted from DCM: water. Compound **2** has been isolated from column chromatography with silica gel (DCM) (230-400 mesh) (yield: 34%).

*Spectral data of* ***2***: MS (MALDI-TOF) (SA) m/z Calc.: 398.20; found: 398.07 [M]^+^; 378.62 [M-F]^+^ (Fig S4). ^1^H NMR (500 MHz, CDCl_3_, 298 K, δ ppm) 7.16 (d, *J*= 8.4 Hz, 2H, Ar-CH), 7.01 (d, *J*= 8.4 Hz, 2H, Ar-CH), 5.97 (s, 2H, -CH), 4.18 (t, *J*= 6.0 Hz, 2H, -OCH_2_), 3.91 (t, *J*= 5.7 Hz, 2H, -CH_2_O), 2.55 (s, 6H, -CH_3_,), 2.09 (m, 2H, -CH_2_-), 1.43 (s, 6H, -CH_3_) (Fig S5). ^13^C NMR (126 MHz, CDCl_3_, 298 K, δ ppm) δ 159.38, 155.28, 143.12, 141.78, 131.83, 129.24, 127.23, 121.09, 115.05, 77.01, 65.64, 60.24, 32.03, 14.59 (Fig S6).

**Synthesis of compound 3**

4-Bromo-1,8-naphthalic anhydride (3.00 g, 10.8 mmol) was dissolved in ethanol (80 mL) in a 250 mL round-bottom reaction flask. n-Butylamine (0.79 g, 21.6 mmol) was added to the solution, and the reaction mixture was stirred at 80 °C under reflux for 6 hours using a magnetic stirrer. The progress of the reaction was monitored by thin-layer chromatography (TLC). After completion, the reaction mixture was cooled to room temperature, and water (200 mL) was added. The resulting precipitate was collected by filtration through a G4 glass filter. The solid was washed sequentially with water (50 mL), a mixture of EtOH/H₂O (25 mL:25 mL), and ethanol (50 mL). The product was dried under reduced pressure to afford compound **3** as a white solid (3.43 g, 95% yield).

*Spectral data of* ***3***: MS (MALDI-TOF) (CHCA) m/z Calc.: 332.20; found: 332.409 [M]^+^ (Fig S7).^1^H NMR (500 MHz, CDCl_3_, 298 K, δ ppm) 8.66 (d, *J*= 7.2 Hz, 1H, Ar-CH), 8.58 (d, *J*= 8.5 Hz, 1H, Ar-CH), 8.42 (d, *J*= 7.8 Hz, 1H, Ar-CH), 8.05 (d, *J*= 7.9 Hz, 1H), 7.85 (t, *J*= 7.90 Hz, 1H, Ar-CH), 4.17 (t, *J*= 7.60 Hz, 2H, -NCH_2_), 1.75-1.69 (m, 2H, -CH_2_-), 1.46-1.41 (m, 2H, -CH_2_-), 0.98 (t, *J*= 7.4 Hz, 3H, -CH_3_) (Fig S8). ^13^C NMR (126 MHz, CDCl_3_, 298 K, δ ppm) δ 163.68, 133.25, 132.04, 131.23, 131.12, 130.68, 130.21, 129.06, 128.10, 123.21, 122.34, 40.40, 30.18, 20.38, 13.84 (Fig S9).

**Synthesis of compound** **4**

4-Hydroxybenzaldehyde (0.64 g, 5.25 mmol) was dissolved in DMF (25 mL) in a 50 mL round-bottom flask. Sodium hydride (NaH, 0.22 g, 5.69 mmol, 60%) was added to the solution, and the mixture was stirred at room temperature for 10 min. using a magnetic stirrer. Then, Compound 2 (1.45 g, 4.38 mmol) was added to the reaction mixture. The mixture was stirred under reflux at 80 °C for 16 hours, and the progress was monitored by thin-layer chromatography (TLC). Upon completion, the crude reaction mixture was filtered through a G4 glass filter, and the DMF was removed under reduced pressure using a rotary evaporator. The crude product was purified by column chromatography on silica gel (230–400 mesh) using dichloromethane (DCM) as the eluent, affording compound **4** as a pure yellow solid (0.90 g, 55% yield).

*Spectral data of* ***4***: MS (MALDI-TOF) (DHB) m/z Calc.: 373.41; found: 373.188 [M ]^+^ (Fig S10). ^1^H NMR (500 MHz, CDCl_3_, 298 K, δ ppm) 10.02 (s, 1H, -CHO), 8.67 (d, *J* = 7.2 Hz, 1H, Ar-CH), 8.55 (t, *J* = 9.1 Hz, 2H, Ar-CH), 7.99 (d, *J* = 8.5 Hz, 2H, Ar-CH), 7.79 (t, *J* = 7.8 Hz, 1H, Ar-CH), 7.28 (d, *J* = 8.2 Hz, 2H, Ar-CH), 7.14 (d, *J*= 8.1 Hz, 1H), 4.19 (t, *J* = 7.6 Hz, 2H, -NCH_2_), 1.73 (q, *J* = 7.54 Hz, 2H, -CH_2_), 1.50-1.43 (m, 2H, -CH_2_-), 0.99 (t, *J* = 7.3, Hz, 3H) (Fig S11). ^13^C NMR (126 MHz, CDCl_3_, 298 K, δ ppm) δ 190.62, 164.22, 163.61, 160.98, 157.65, 133.22, 132.46, 132.34, 132.14, 129.89, 128.25, 127.15, 124.57, 123.05, 119.89, 118.56, 113.59, 53.56, 40.38, 30.35, 20.51, 13.97 (Fig S12).


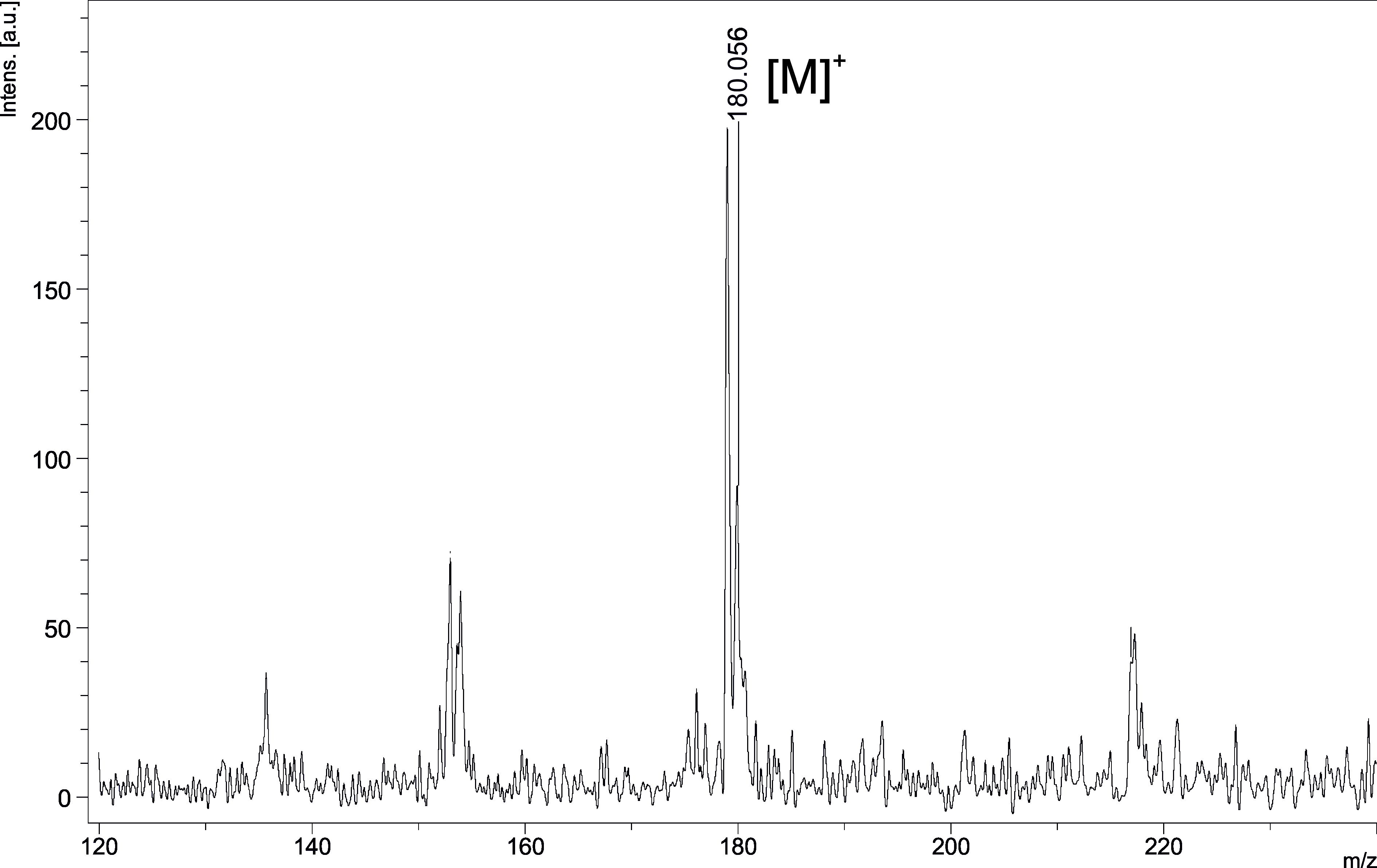


Fig. S1 MALDI-MS spectrum of compound **1**.


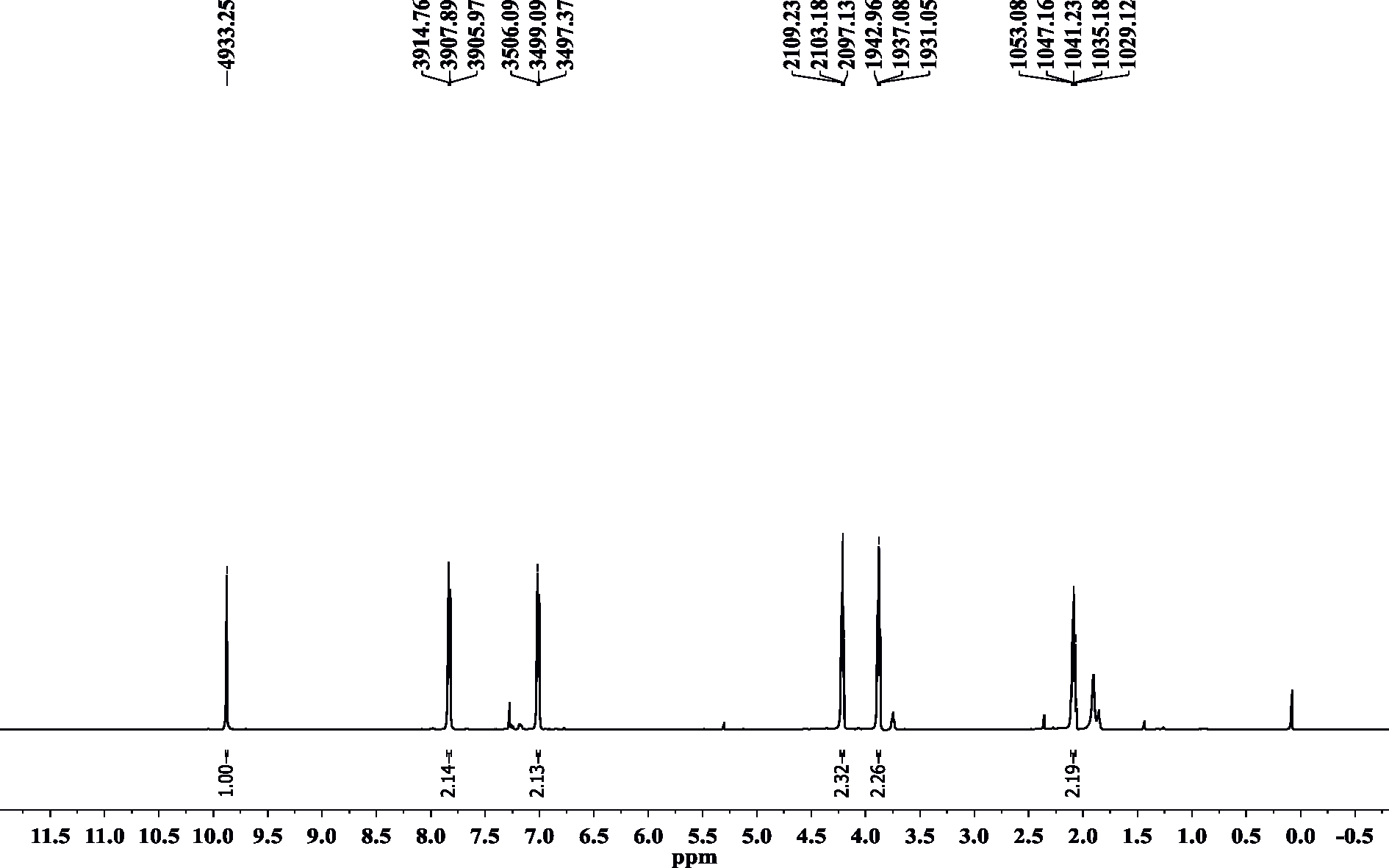


Fig. S2 ^1^H NMR spectrum of compound **1**.


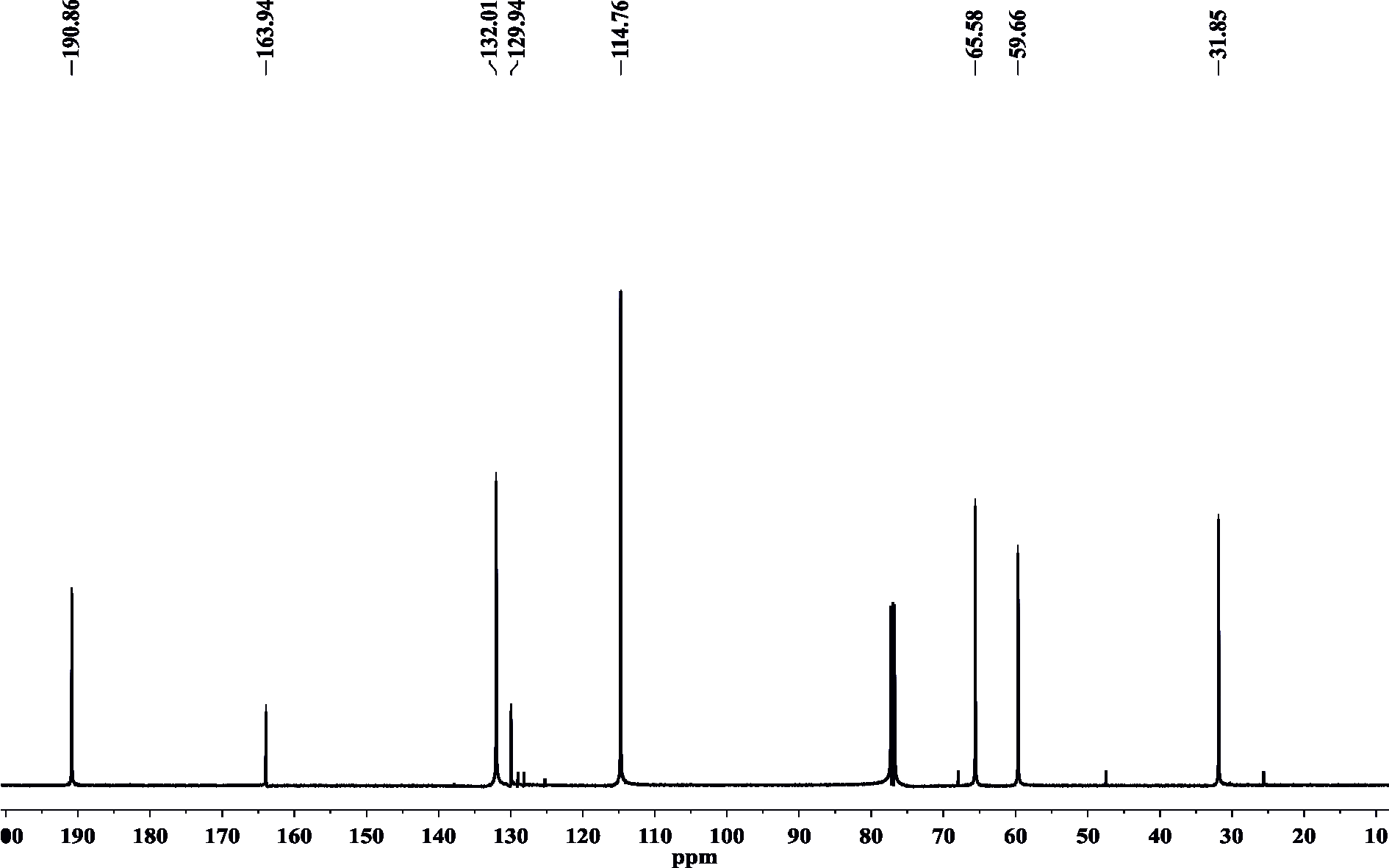


Fig. S3 ^13^C NMR spectrum of compound **1**.


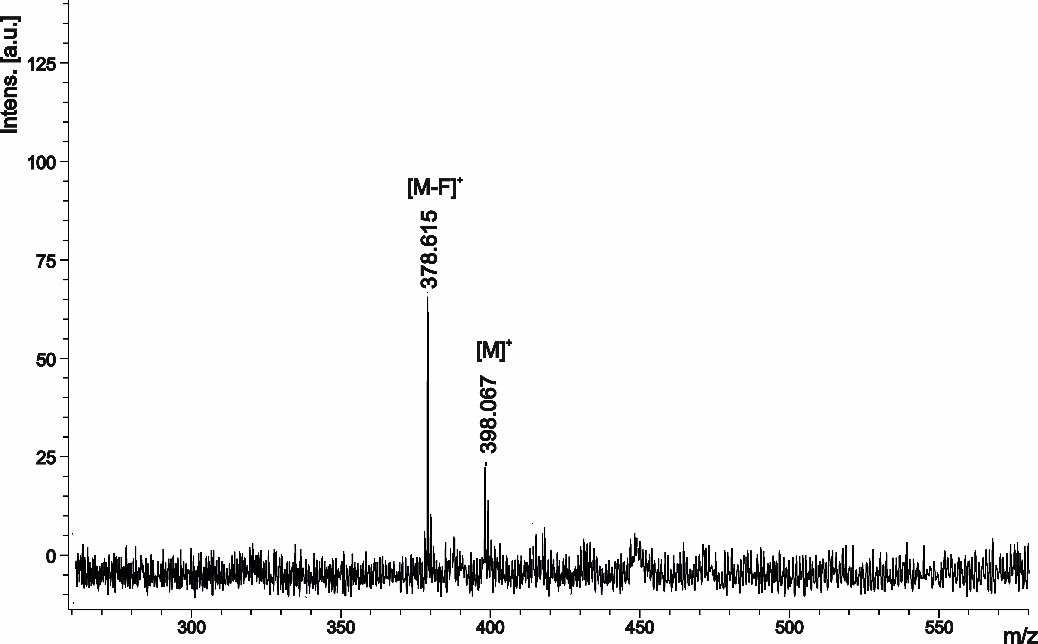


Fig. S4 MALDI-MS spectrum of compound **2**.


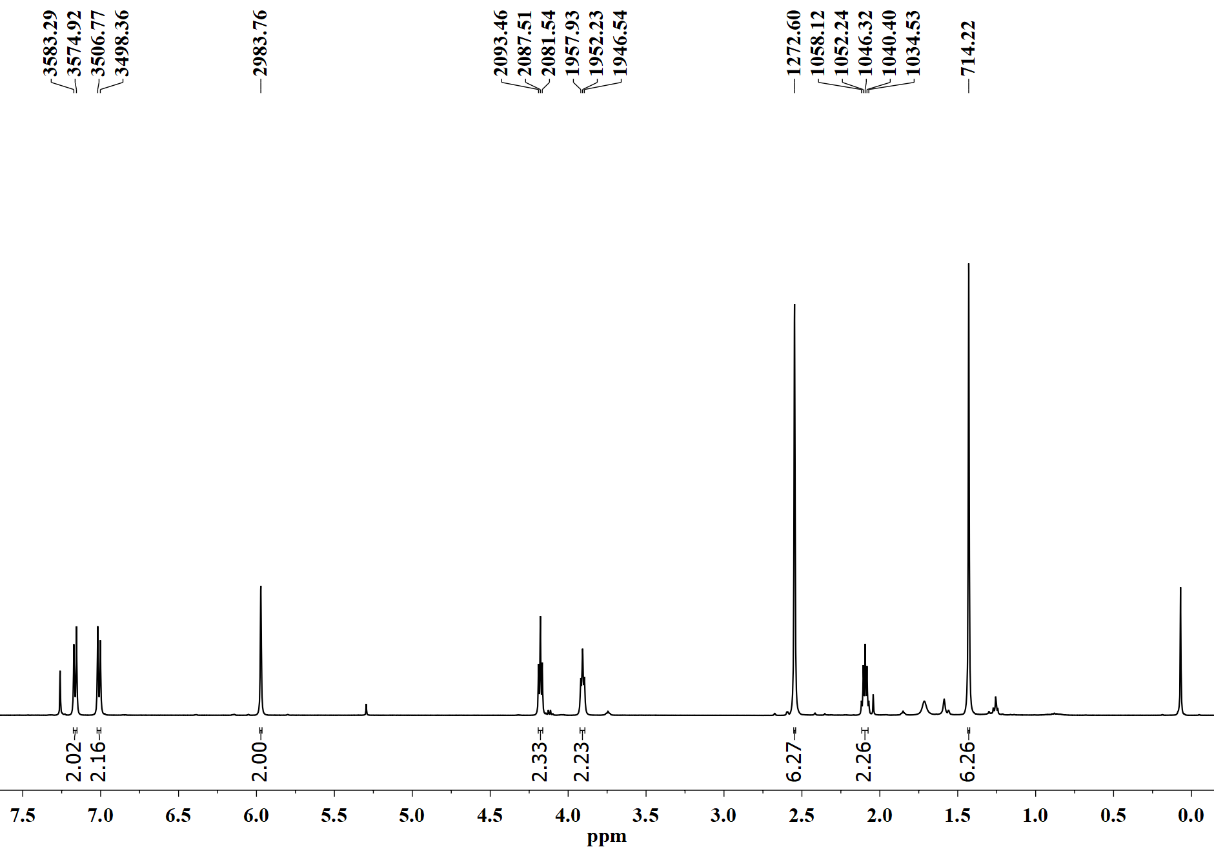


Fig. S5 ^1^H NMR spectrum of compound **2**.


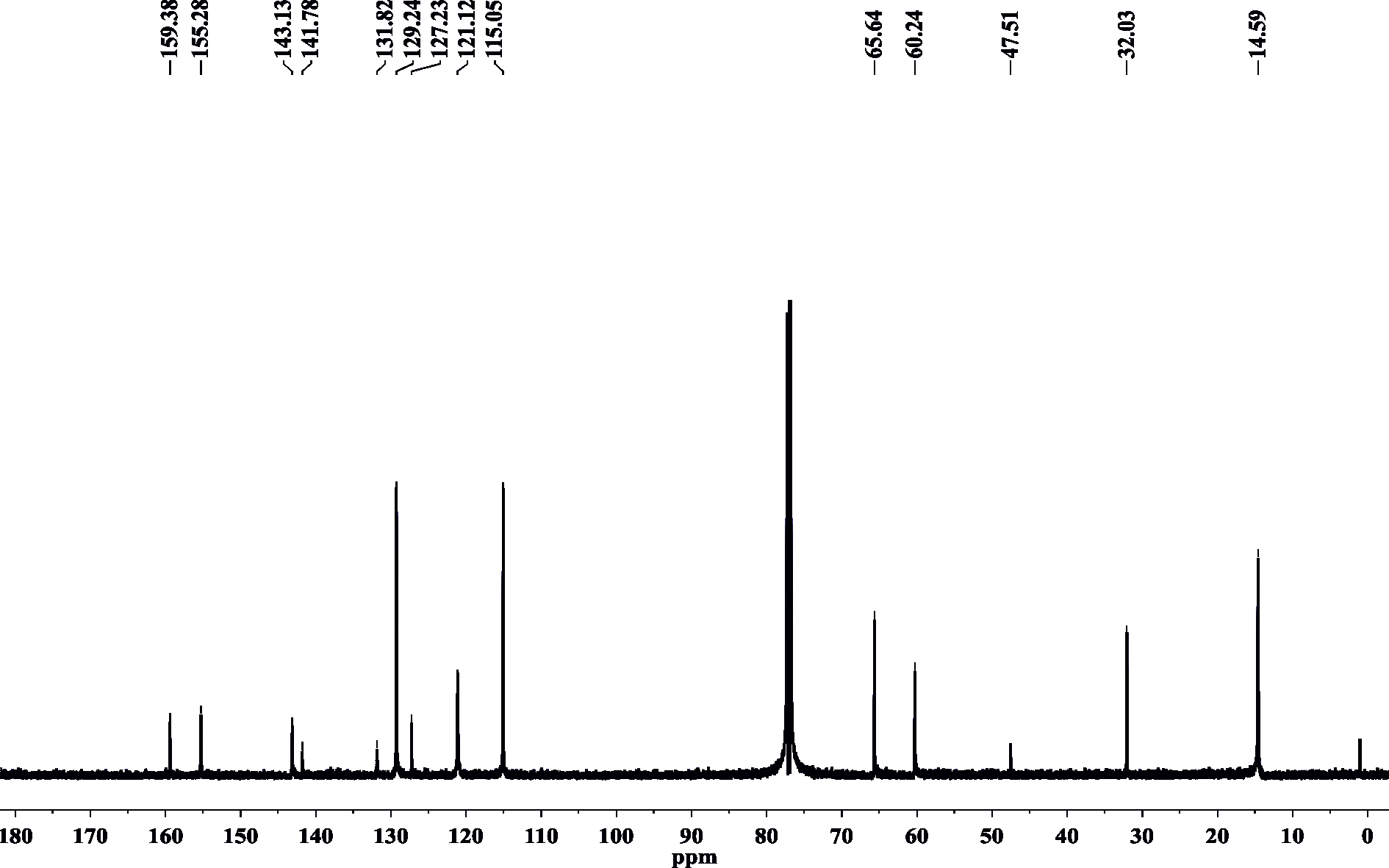


Fig. S6 ^13^C NMR spectrum of compound **2**.


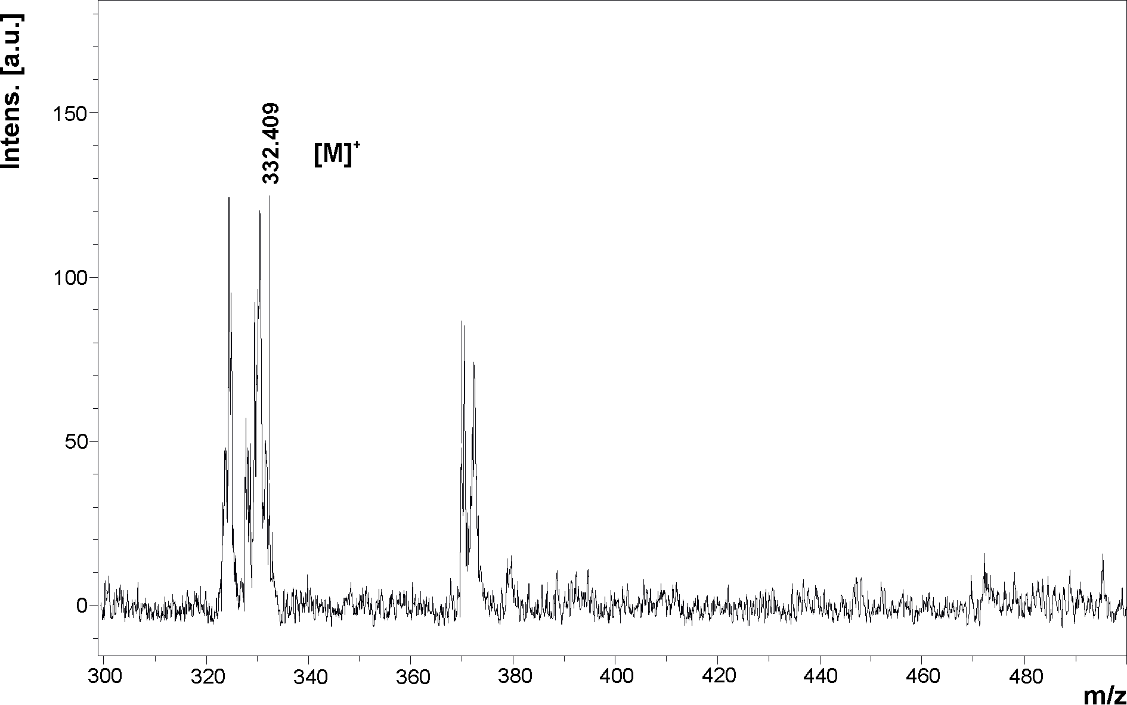


Fig. S7 MALDI-MS spectrum of compound **3**.

Fig. S8 ^1^H NMR spectrum of compound **3**.


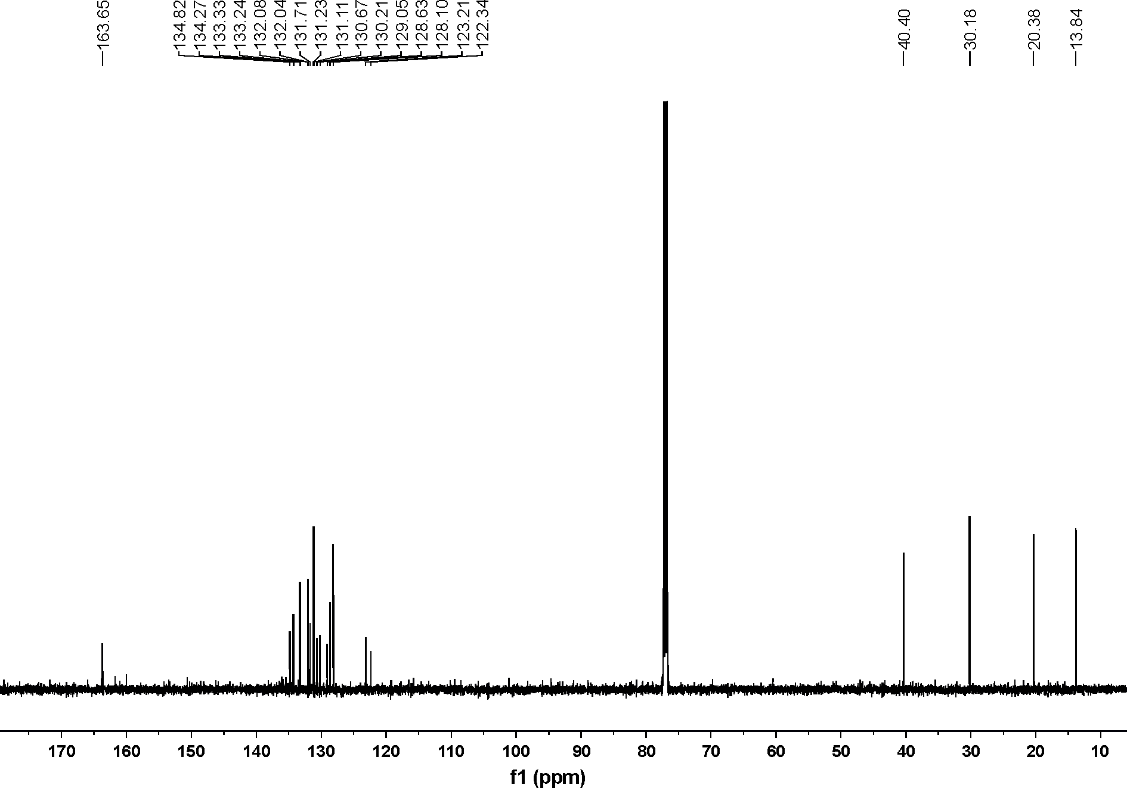


Fig. S9 ^13^C NMR spectrum of compound **3**.


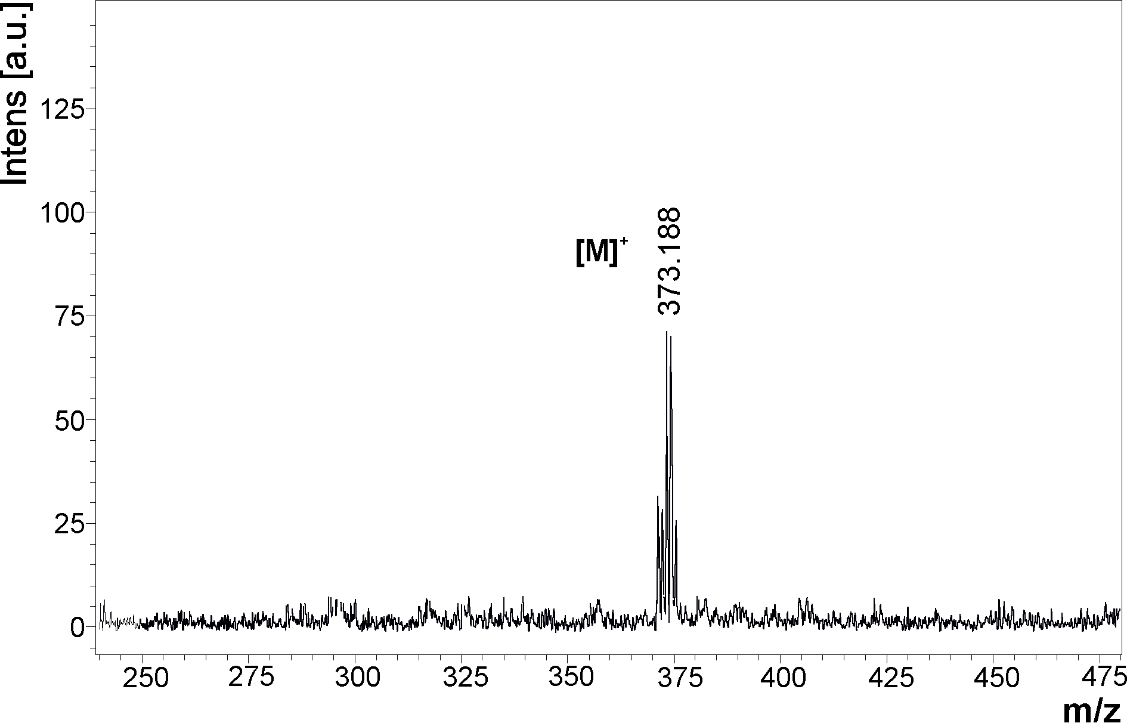


Fig. S10 MALDI-MS spectrum of compound **4**.


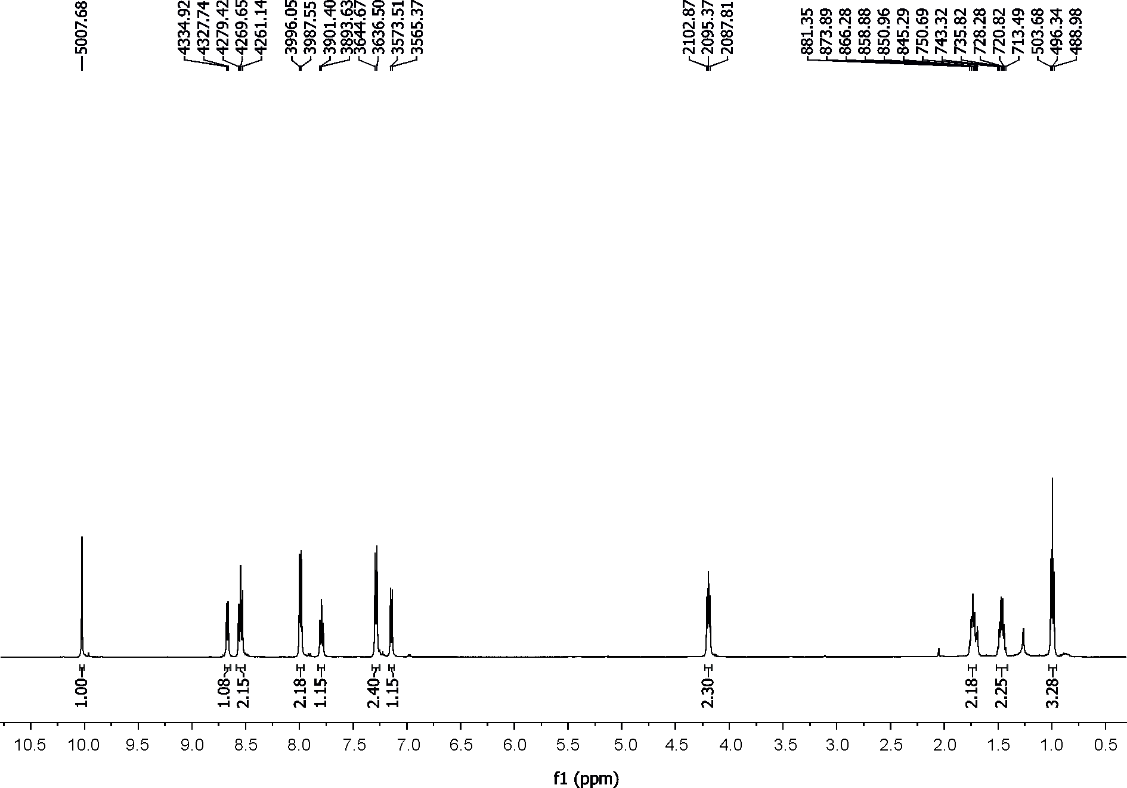


Fig. S11 ^1^H NMR spectrum of compound **4**.


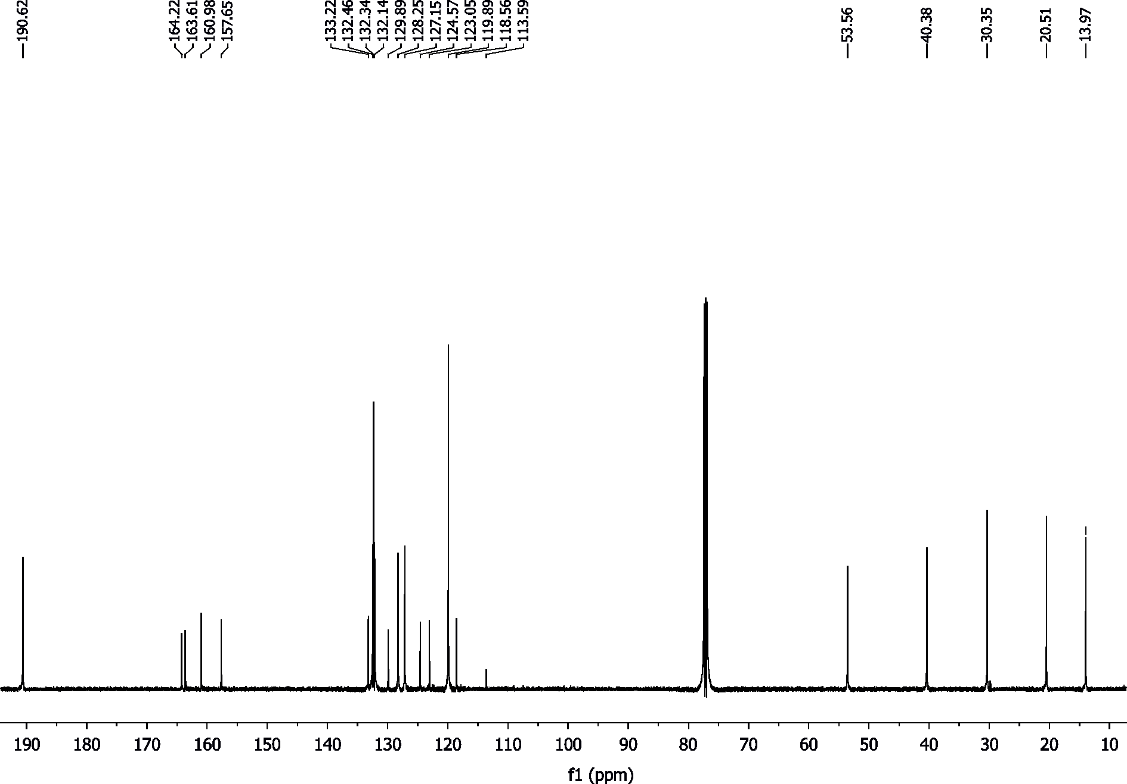


Fig. S12 ^13^C NMR spectrum of compound **4**.


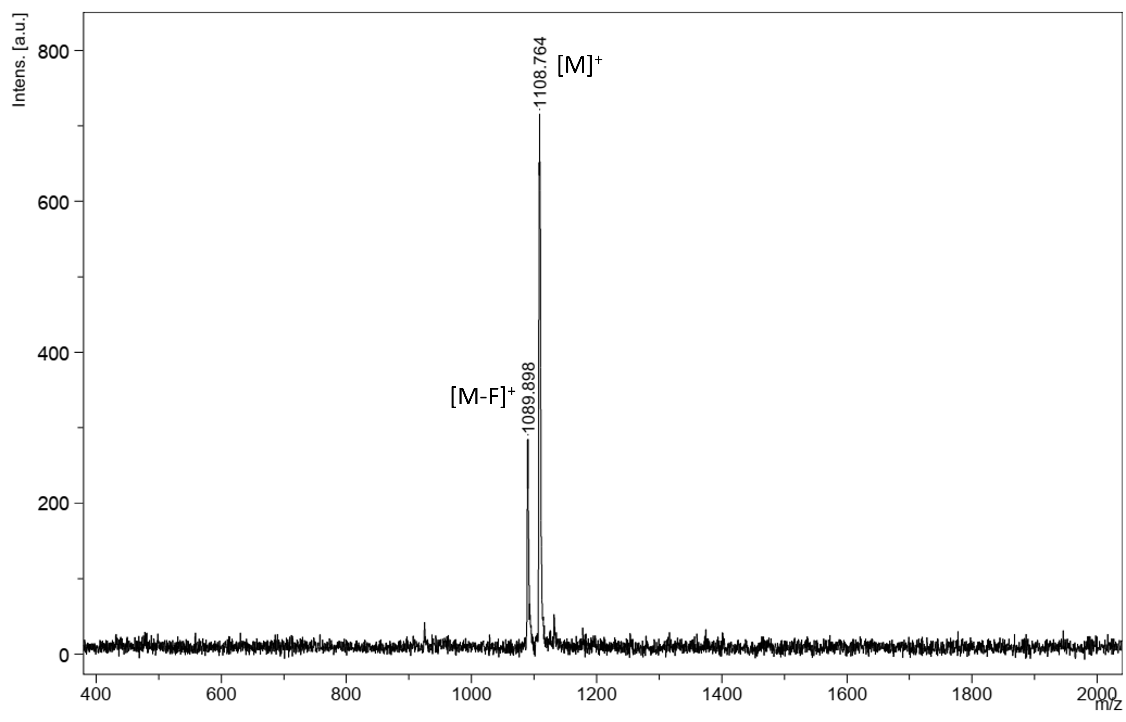


Fig. S13 MALDI-MS spectrum of compound **5**.


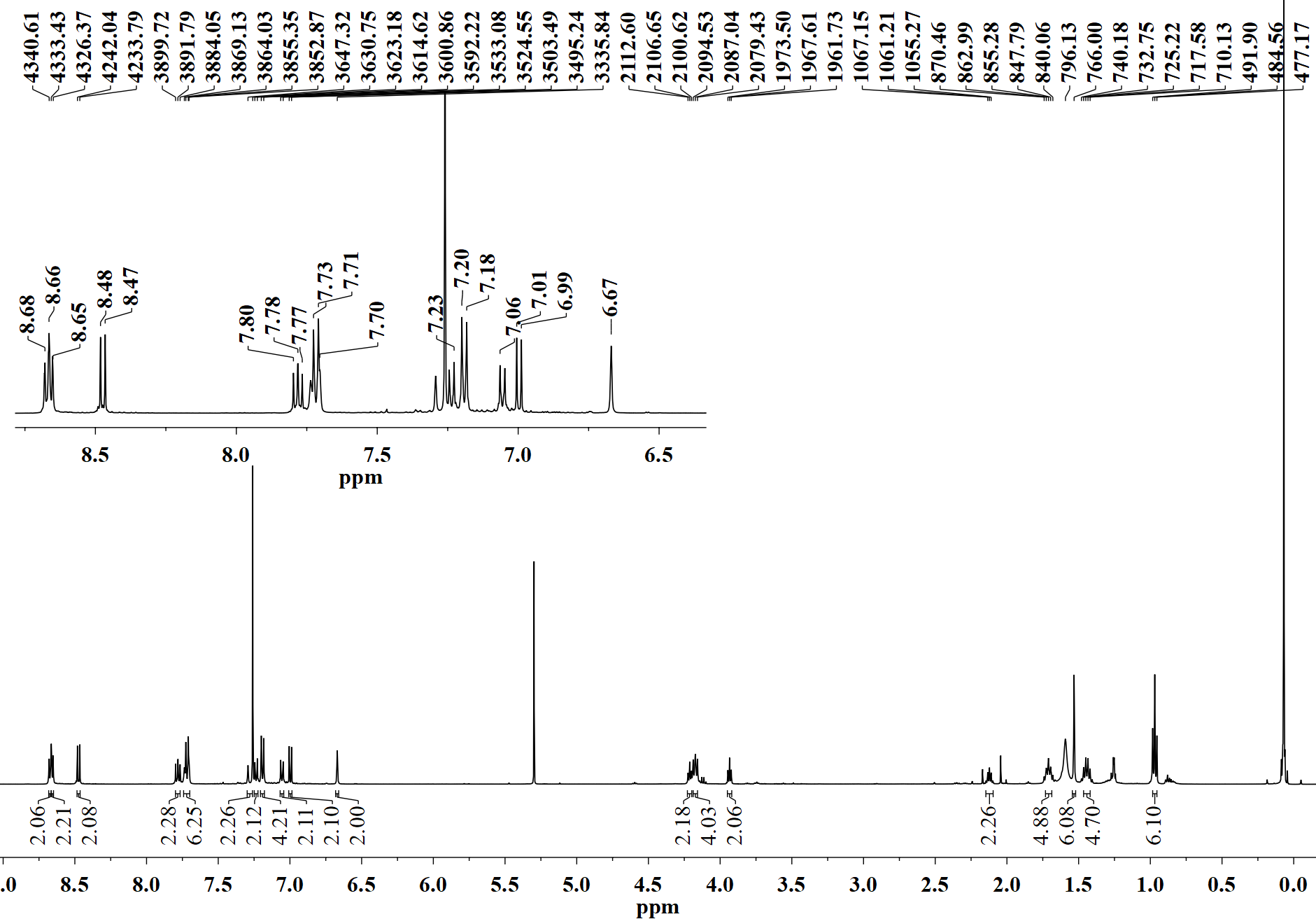


Fig. S14 ^1^H NMR spectrum of compound **5**.


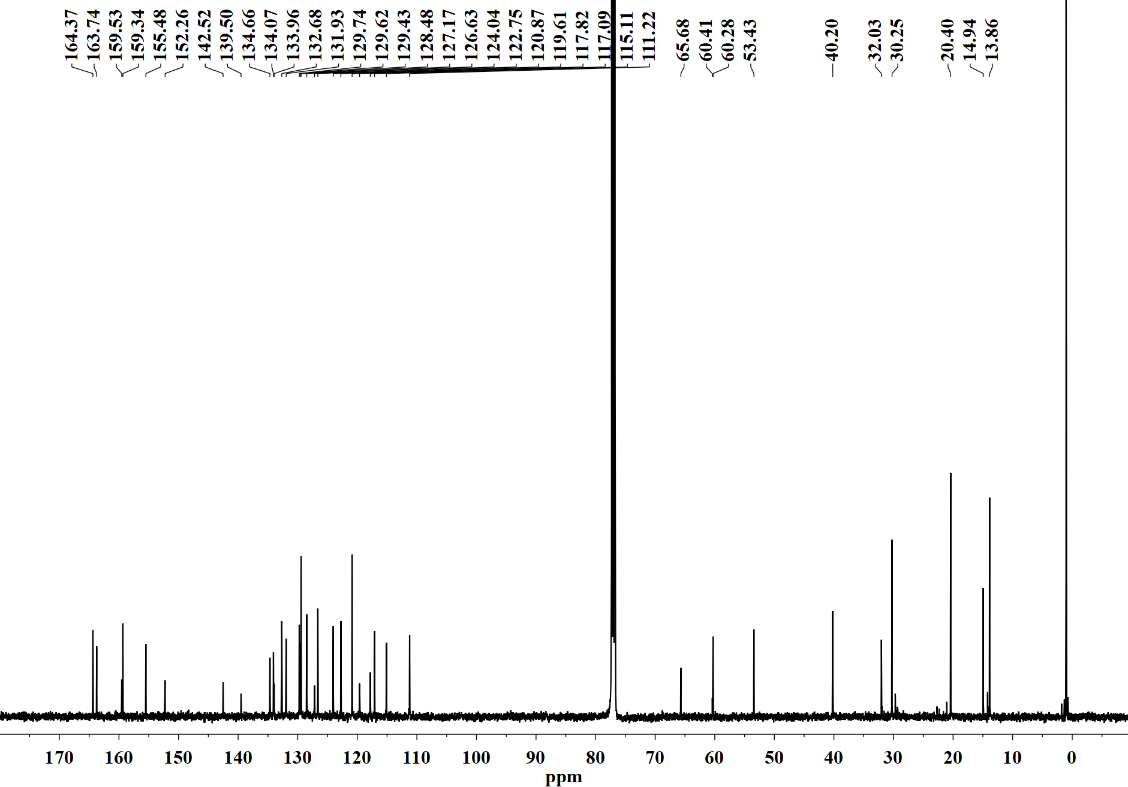


Fig. S15 ^13^C NMR spectrum of compound **5**.


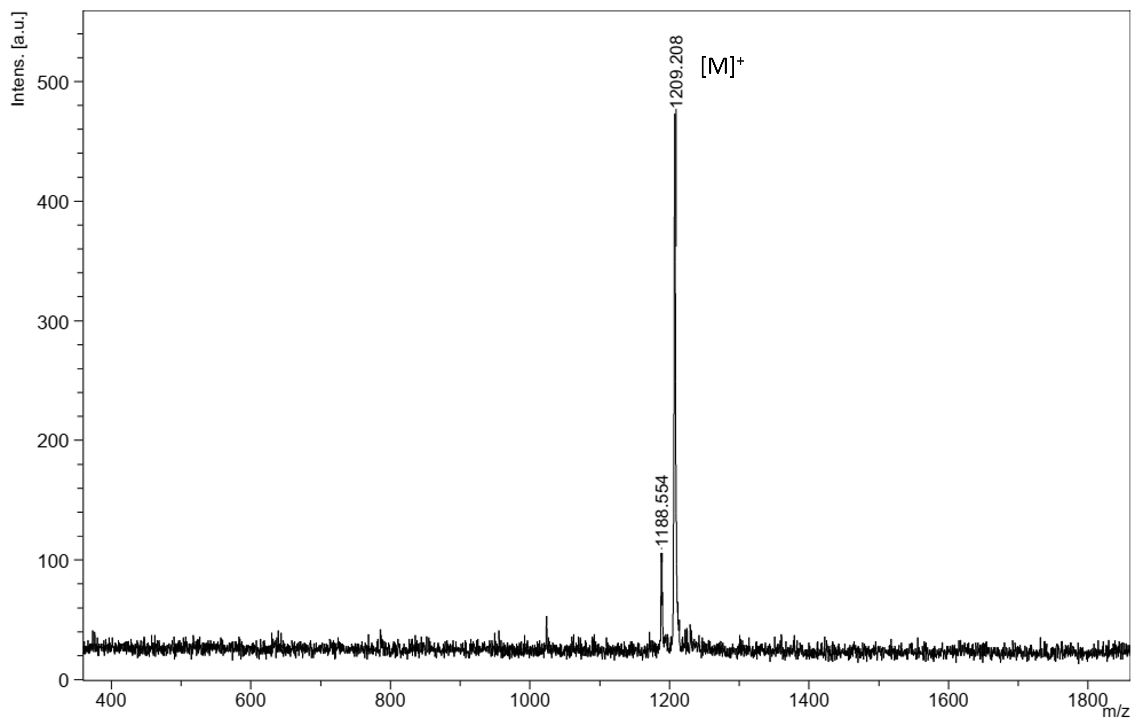


Fig. S16 MALDI-MS spectrum of compound **6**.


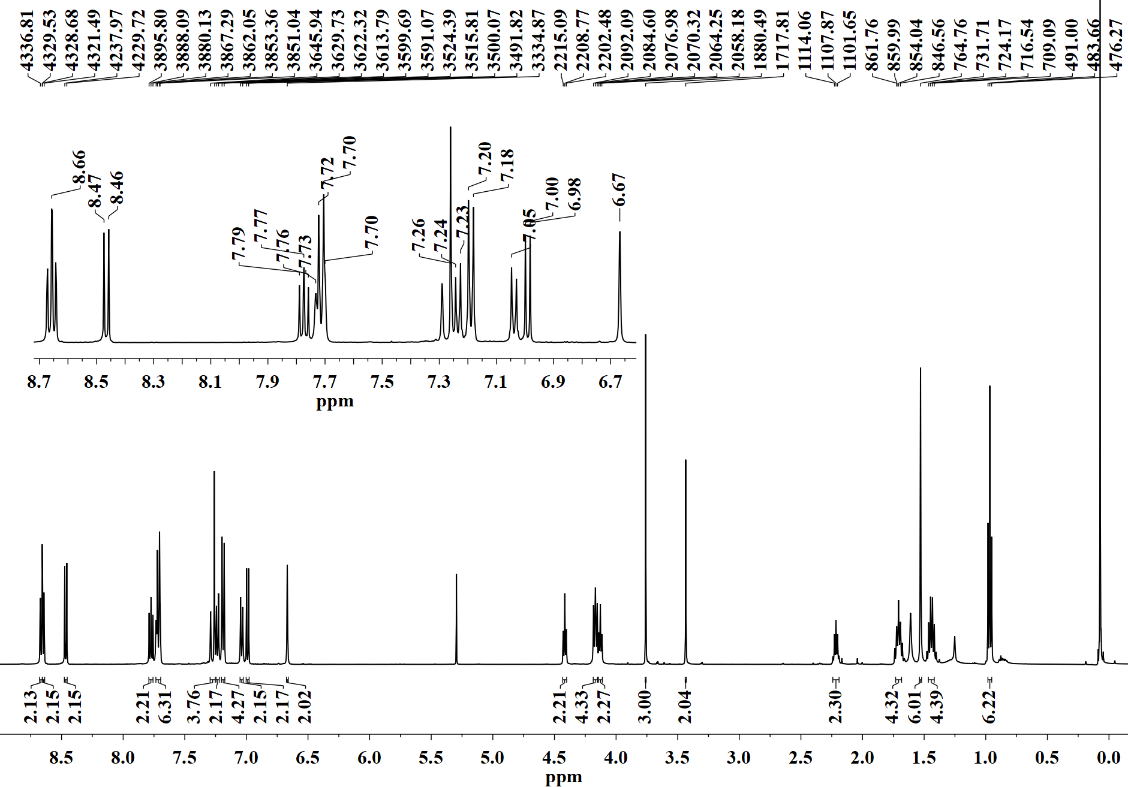


Fig. S17 ^1^H NMR spectrum of compound **6**.


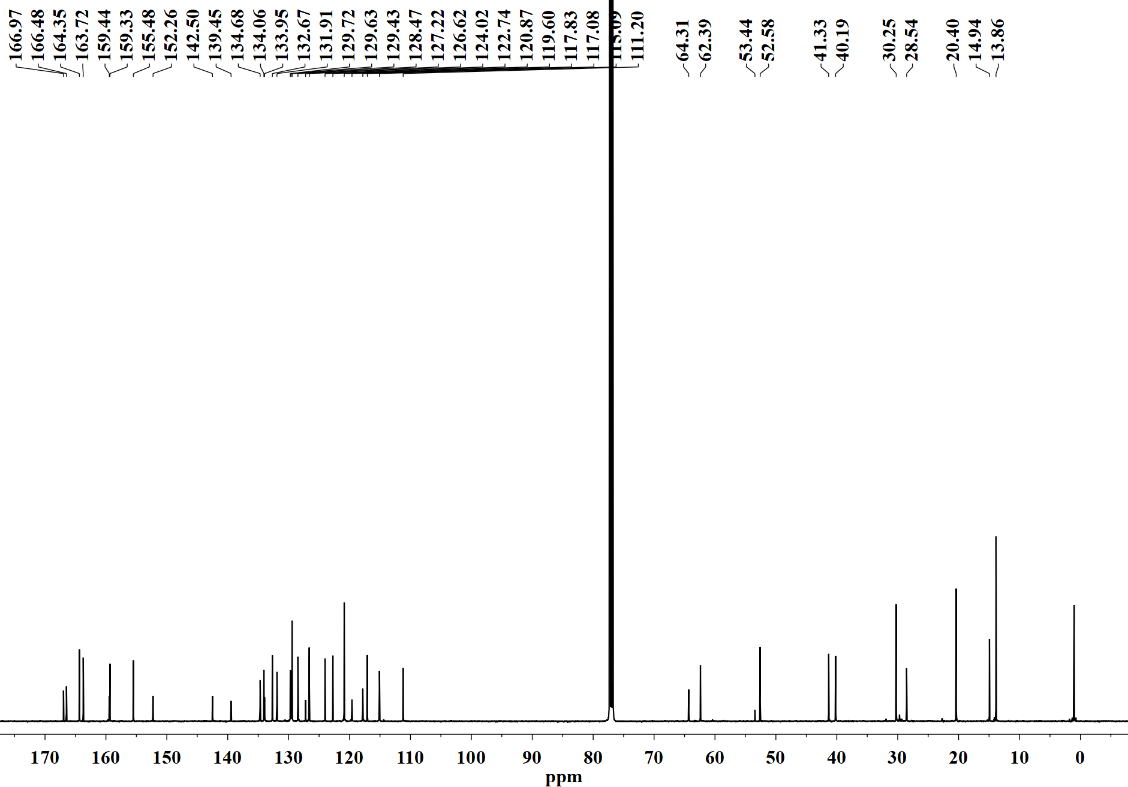


Fig. S18 ^13^C NMR spectrum of compound **6**.

**
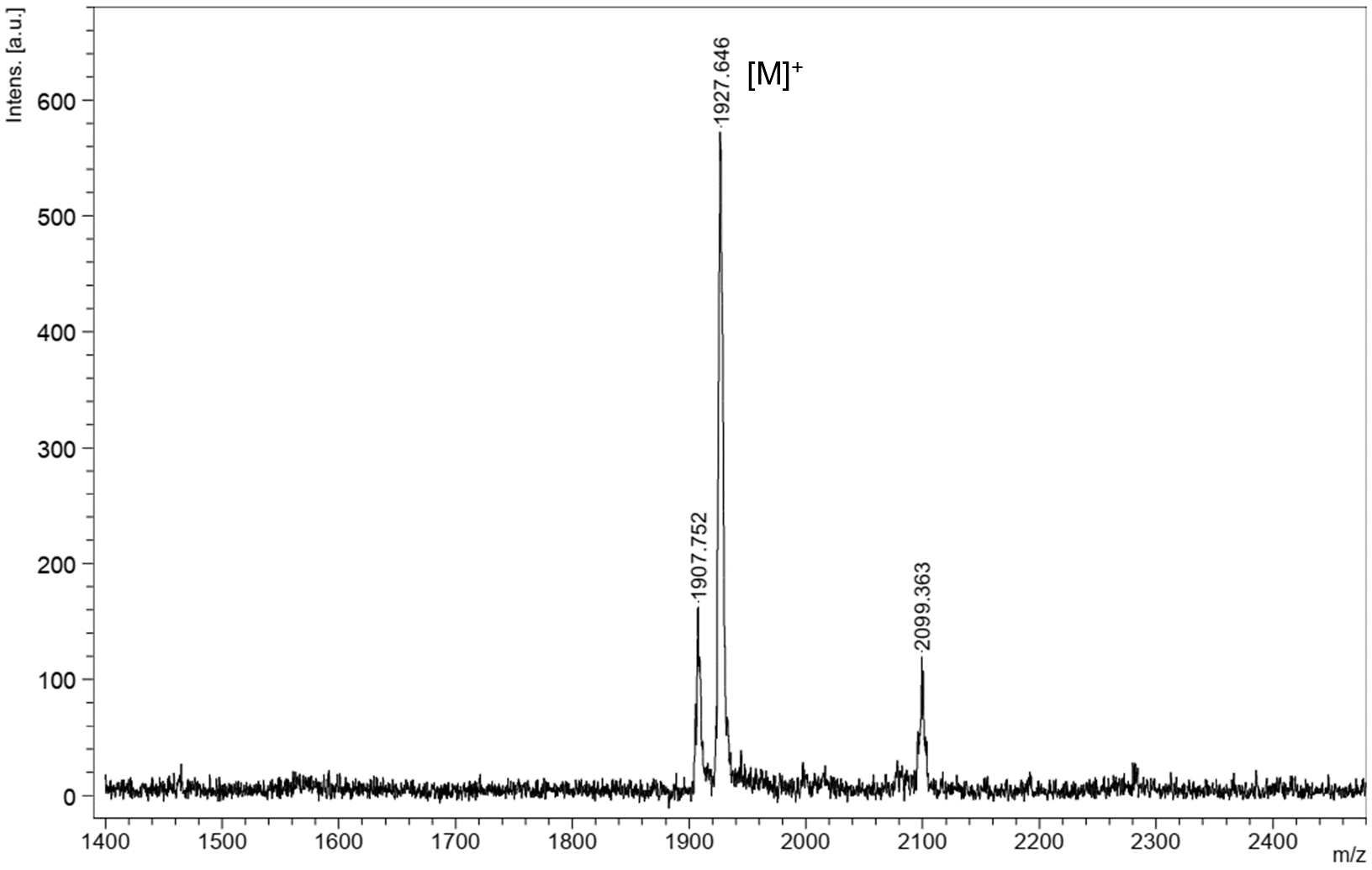
**

Fig. S19 MALDI-MS spectrum of compound **7**.


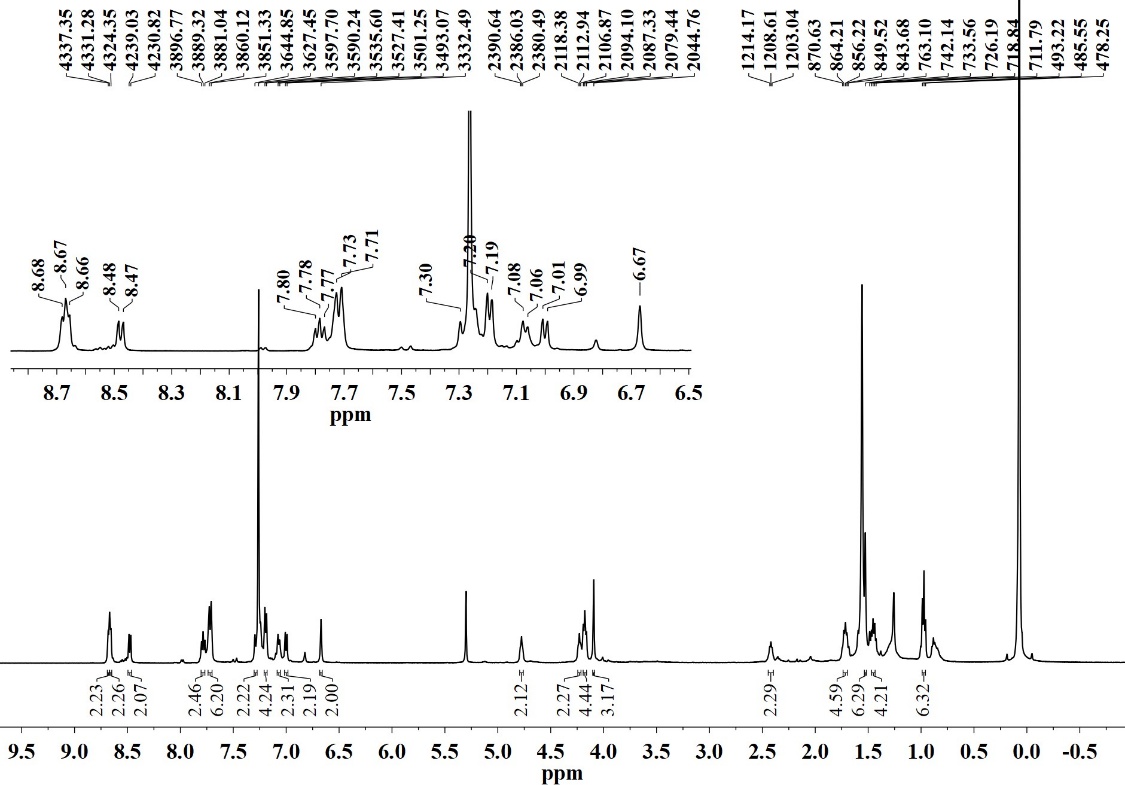


Fig. S20 ^1^H NMR spectrum of compound **7**.


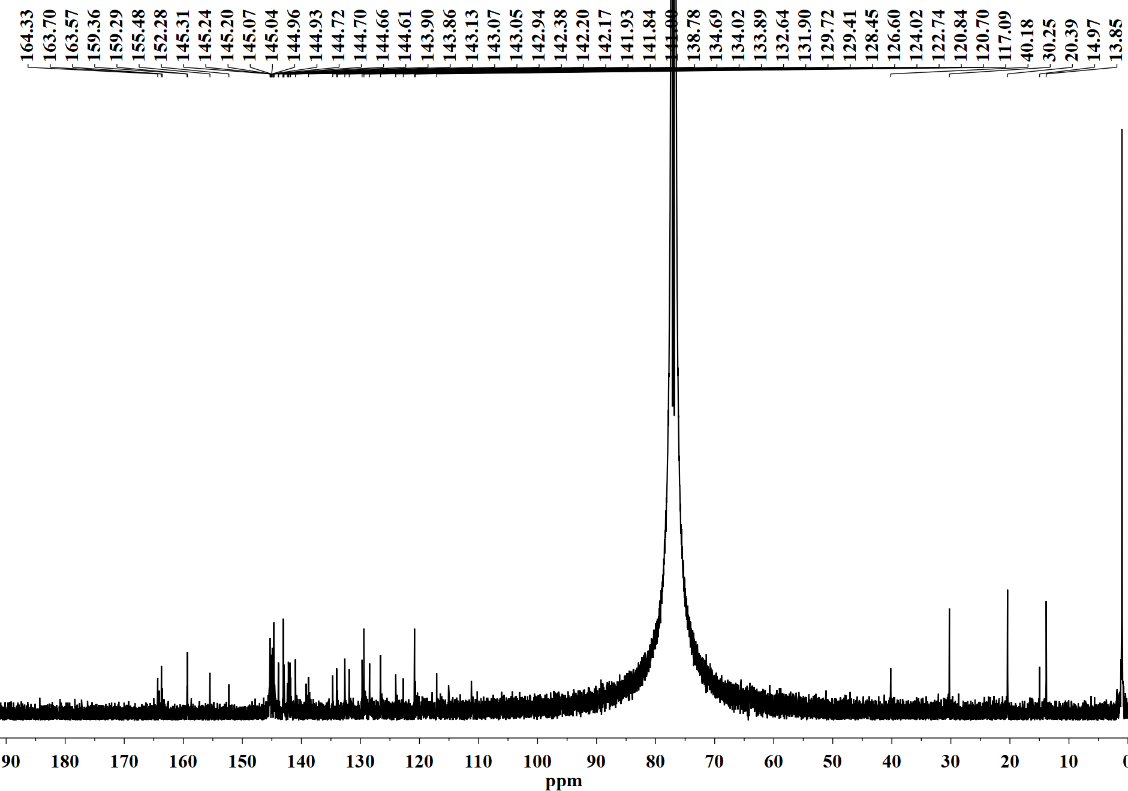


Fig. S21 ^13^C NMR spectrum of compound **7**.


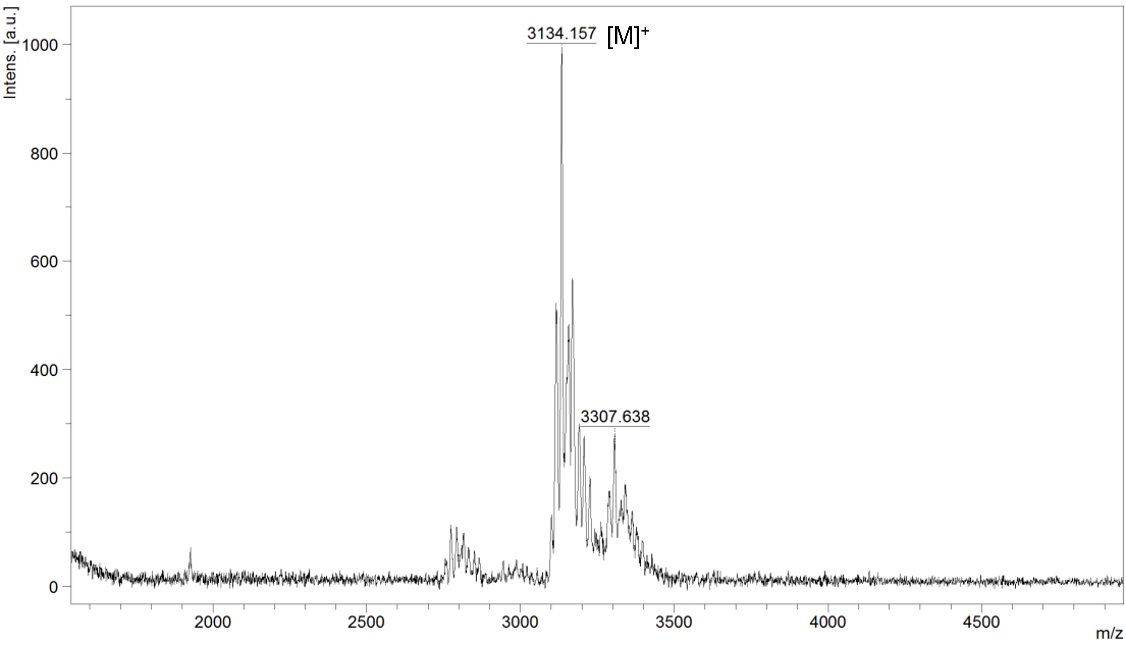


Fig. S22 MALDI-MS spectrum of compound **8**.


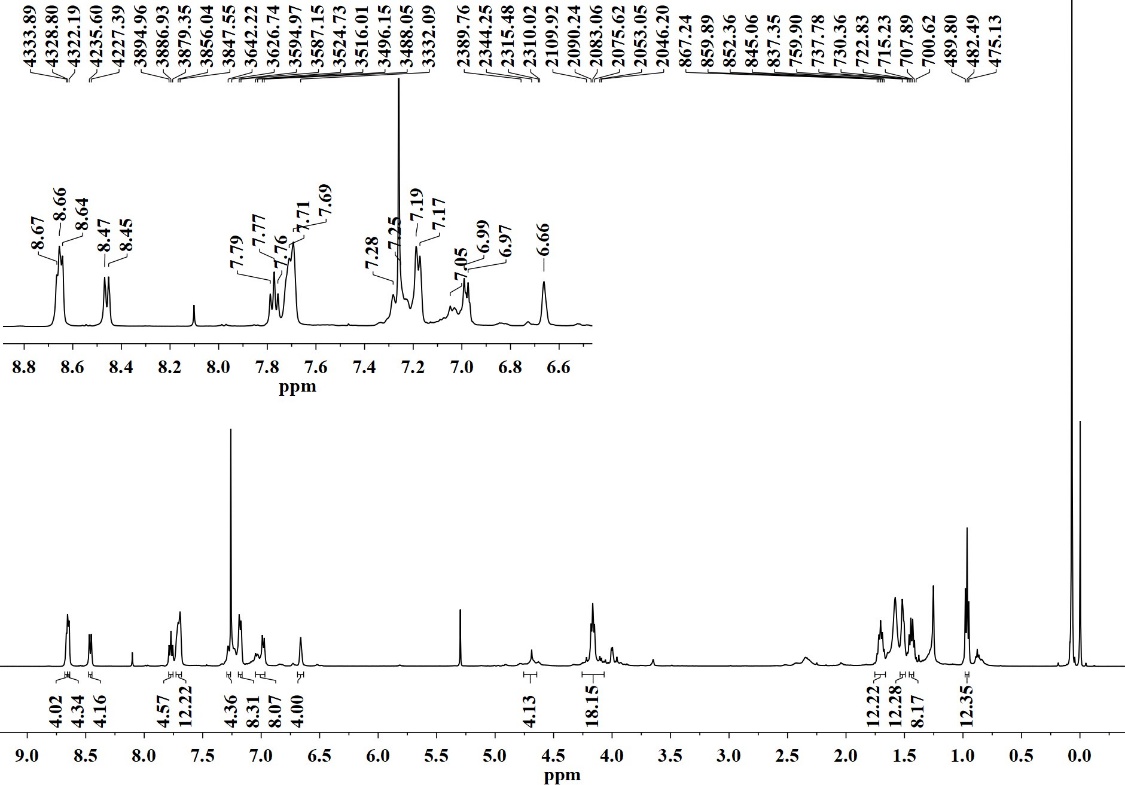


Fig. S23 ^1^H NMR spectrum of compound **8**.


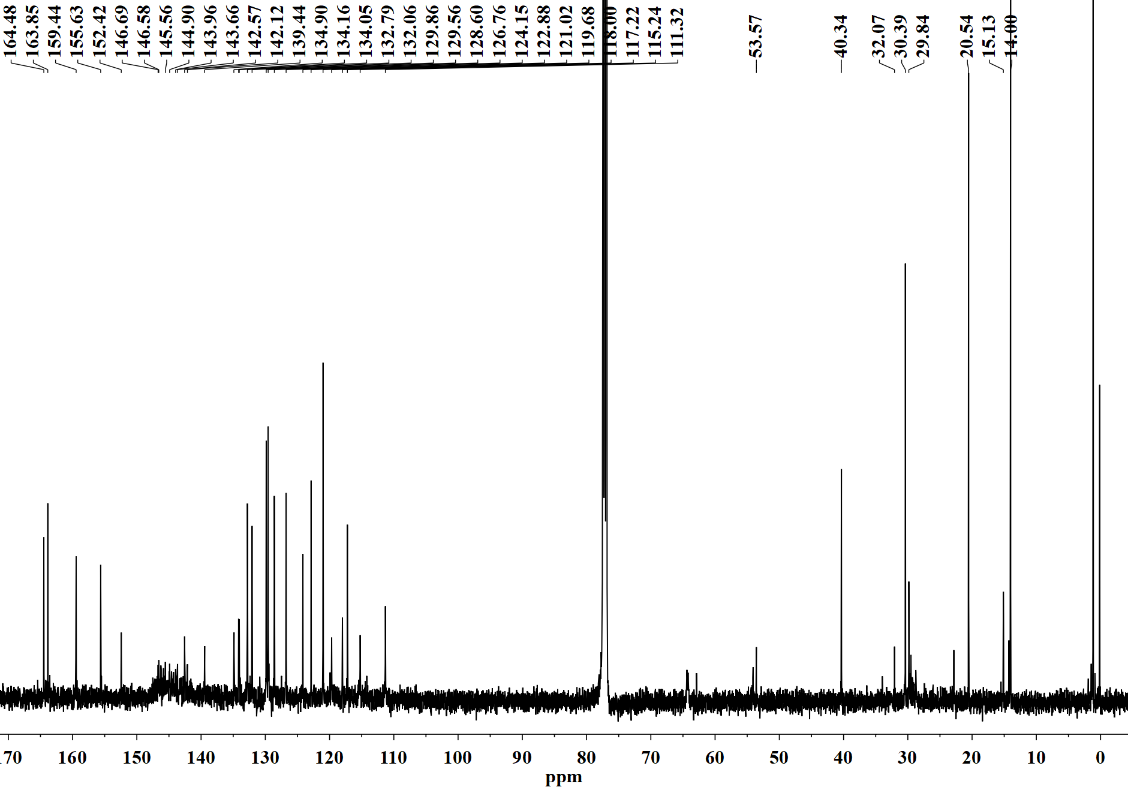


Fig. S24 ^13^C NMR spectrum of compound **8**.


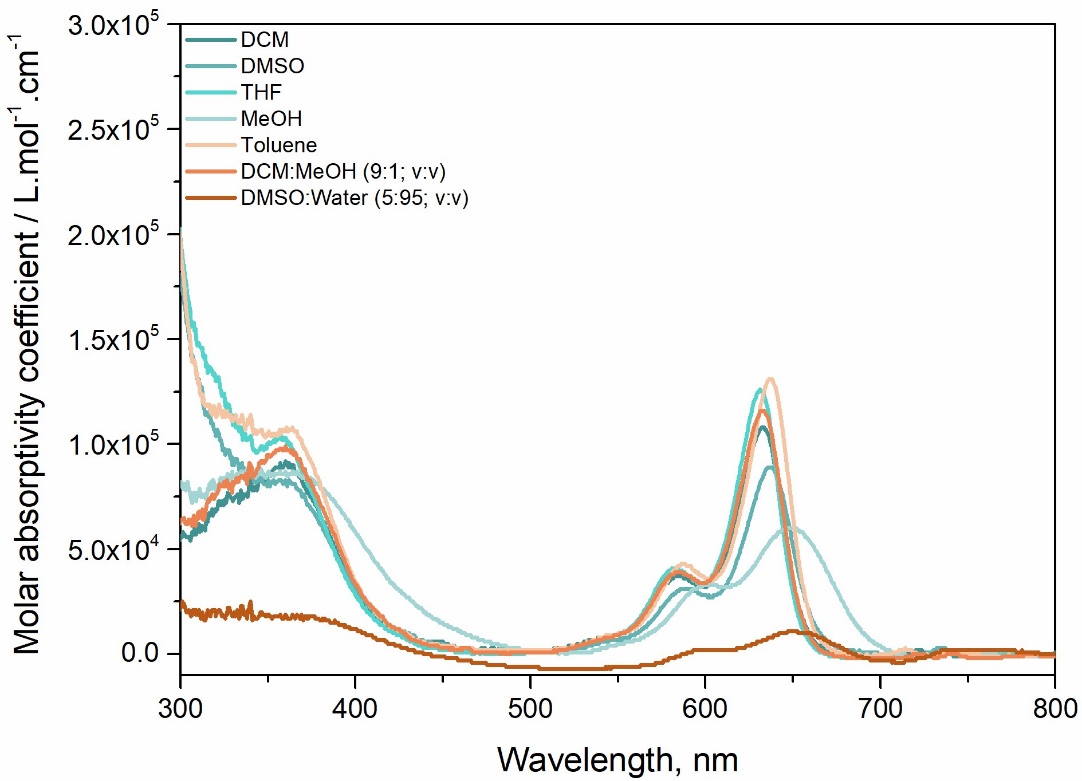


Fig. S25 UV-Vis absorption spectra of compound **7** in different solvents at 1 µM.


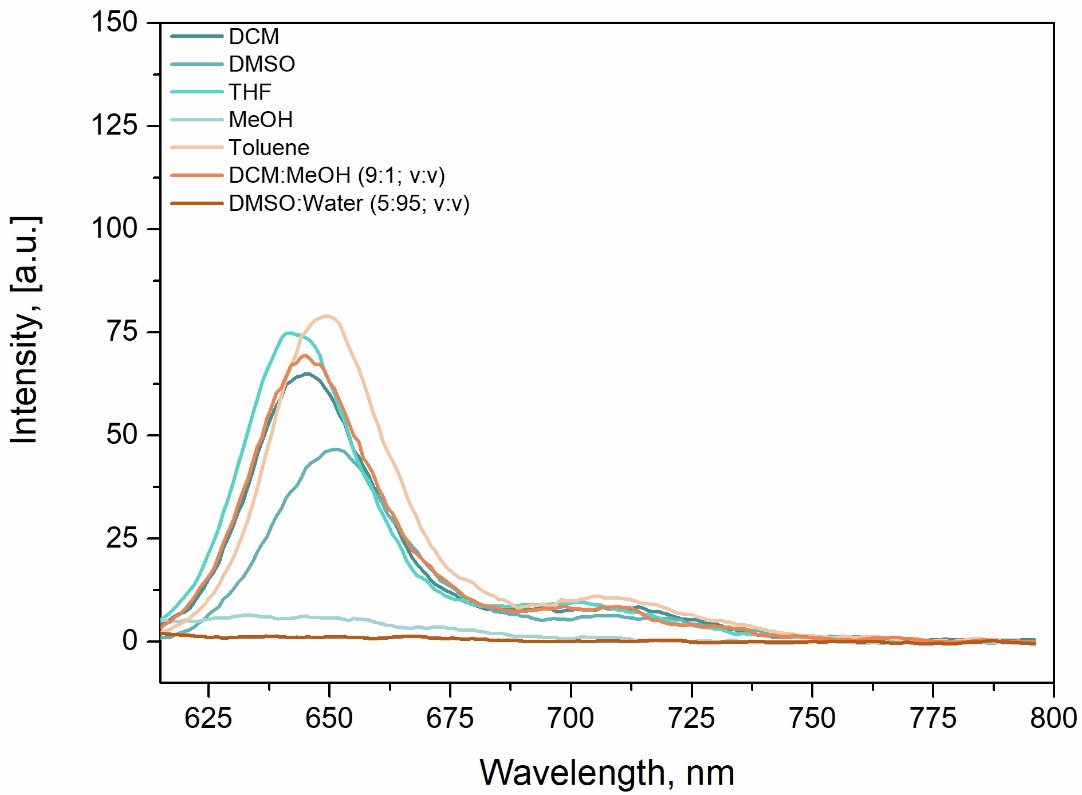


Fig. S26 Fluorescence spectra of compound **7** in different solvents at 1 µM (λ_ex_:610nm).





Fig. S27 UV-Vis absorption spectral change of compound **7** in DCM at varying concentrations.


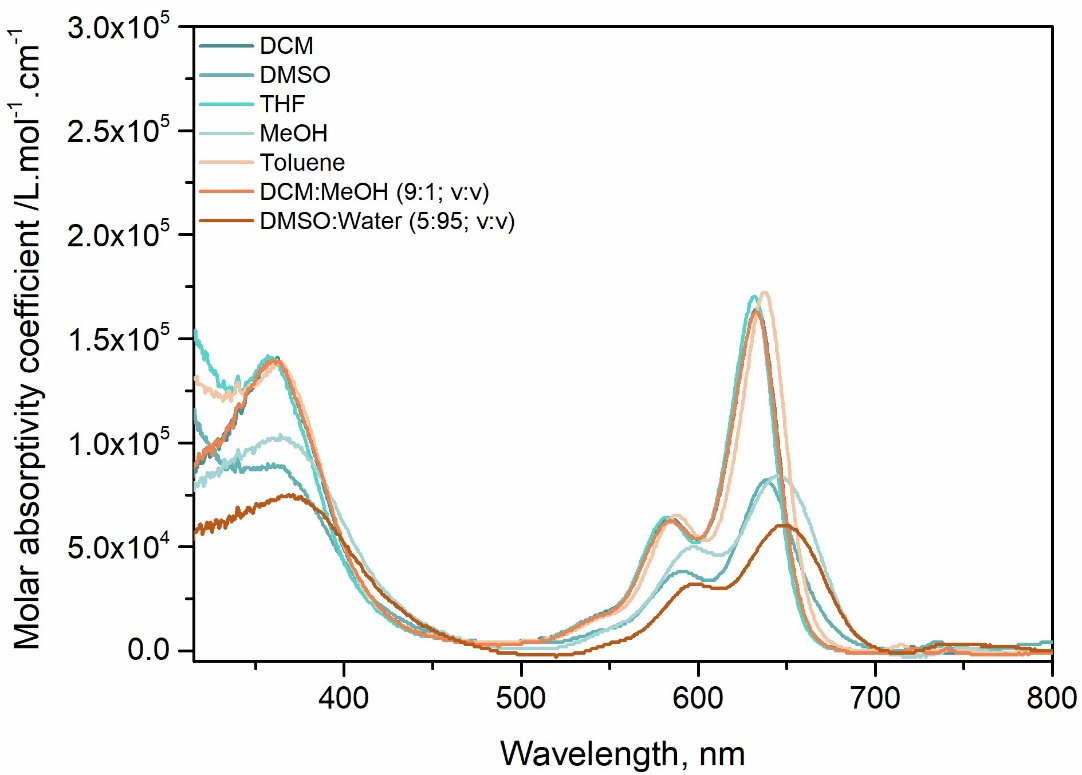


Fig. S28 UV-Vis absorption spectra of compound **8** in different solvents at 1 µM.


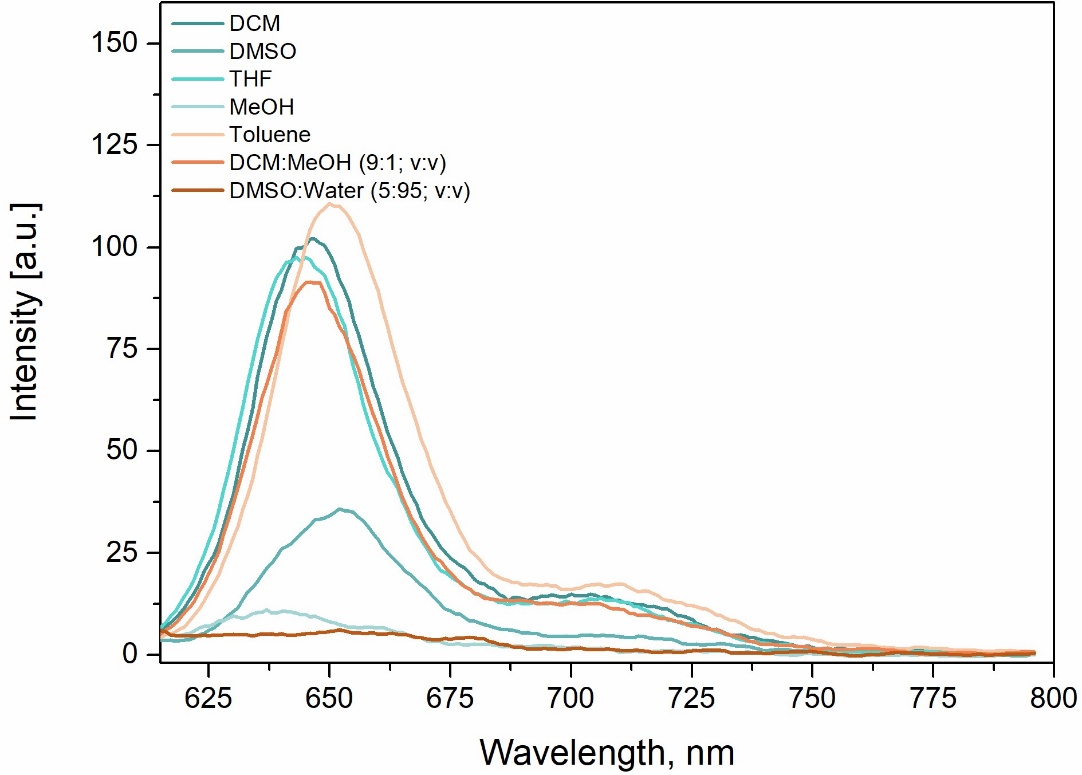


Fig. S29 Fluorescence spectra of compound **8** in different solvents at 1 µM (λ_ex_:610nm).





Fig. S30 UV-Vis absorption spectral change of compound **8** in DCM at varying concentrations.





Fig. S31 Fluorescence lifetime decays of **7** and **8** in DCM (λ_ex_ = 674 nm).


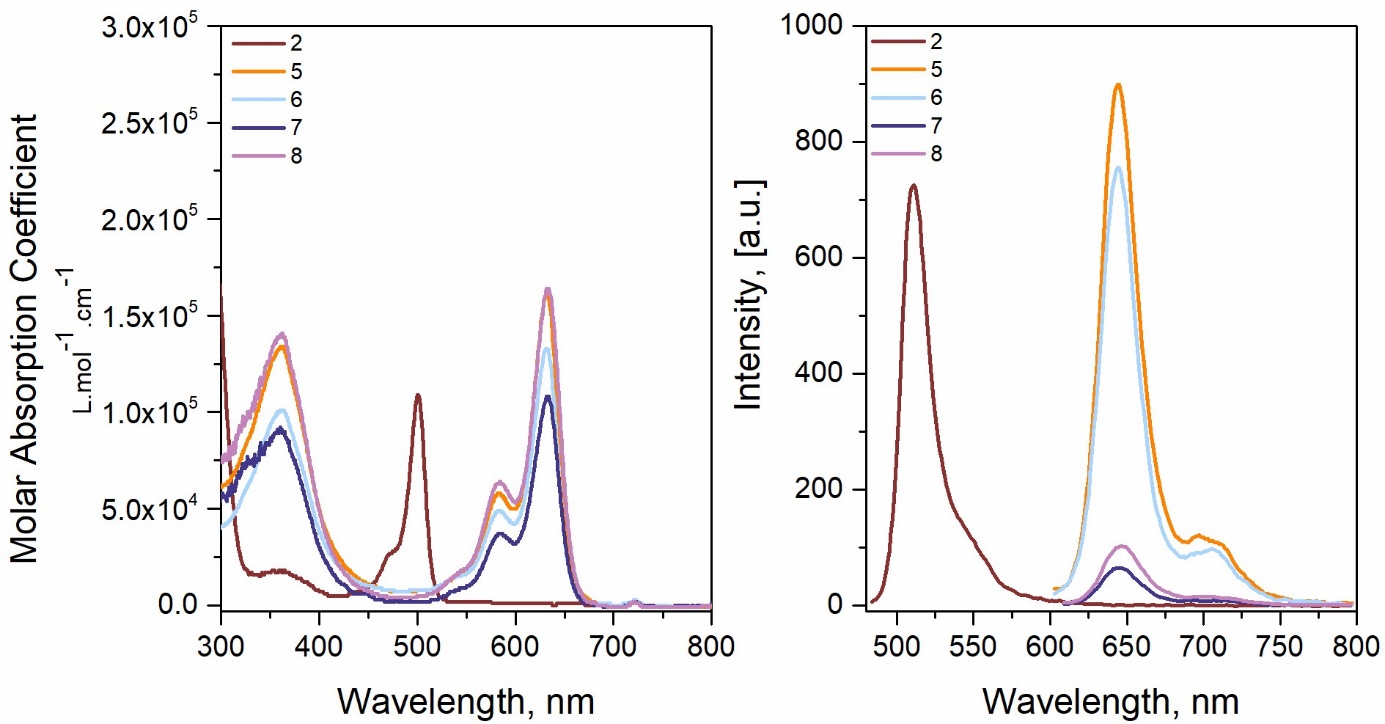


Fig S32. Steady-state UV-vis absorption and fluorescence emission spectra of compounds in DCM.





Fig. S33 Absorbance decrease of DPBF in the presence of MB.


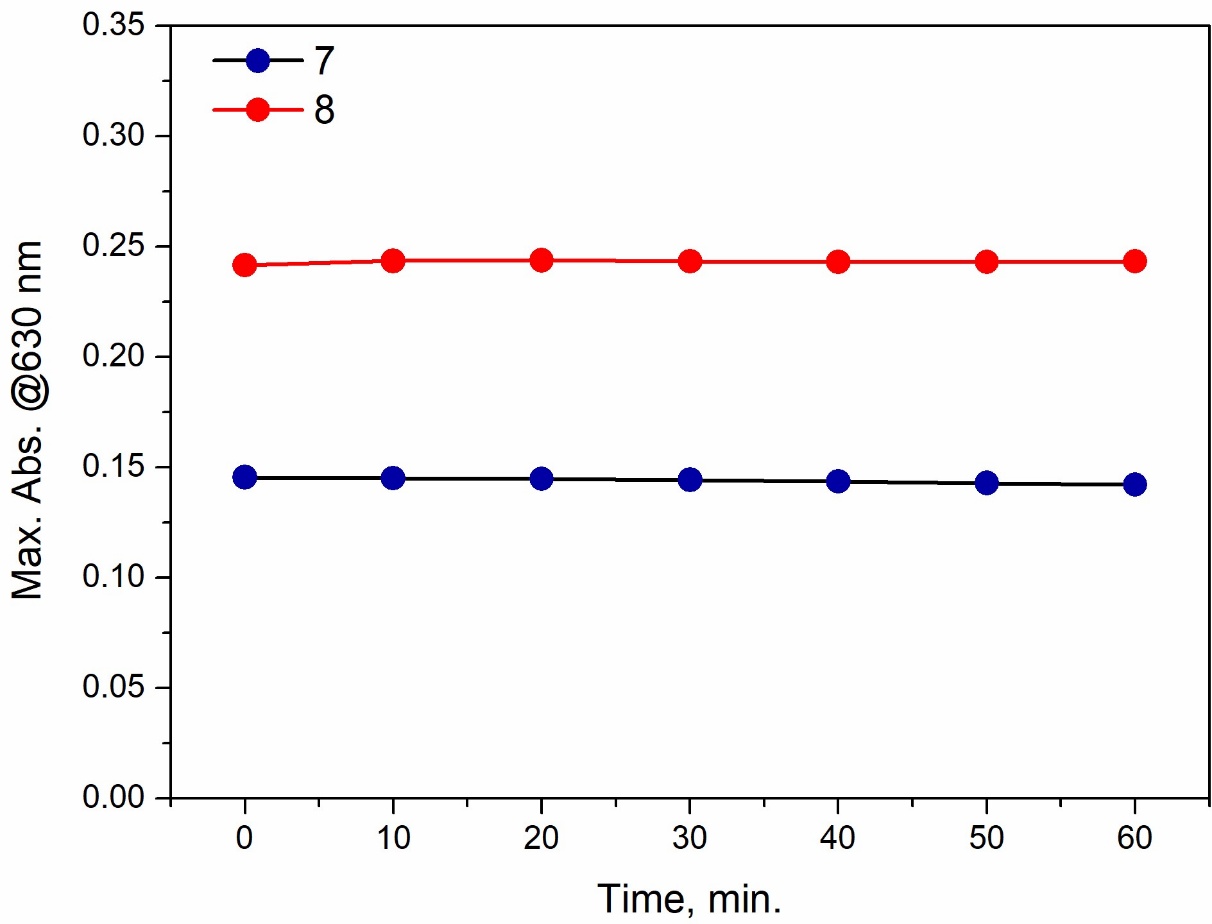


Fig. S34 Photostability of compounds 7 and 8 under light.


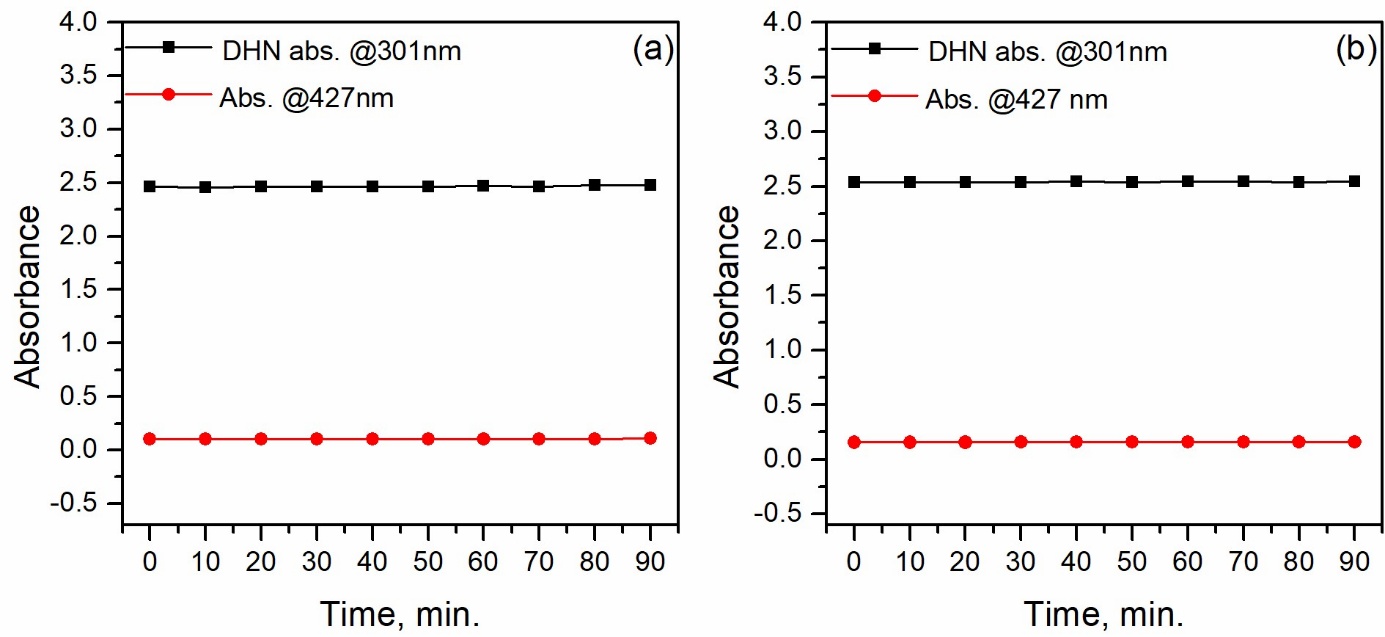


Fig. S35 Dark photostability of DHN and juglone in the presence of (a) compound 7 and (b) compound 8.


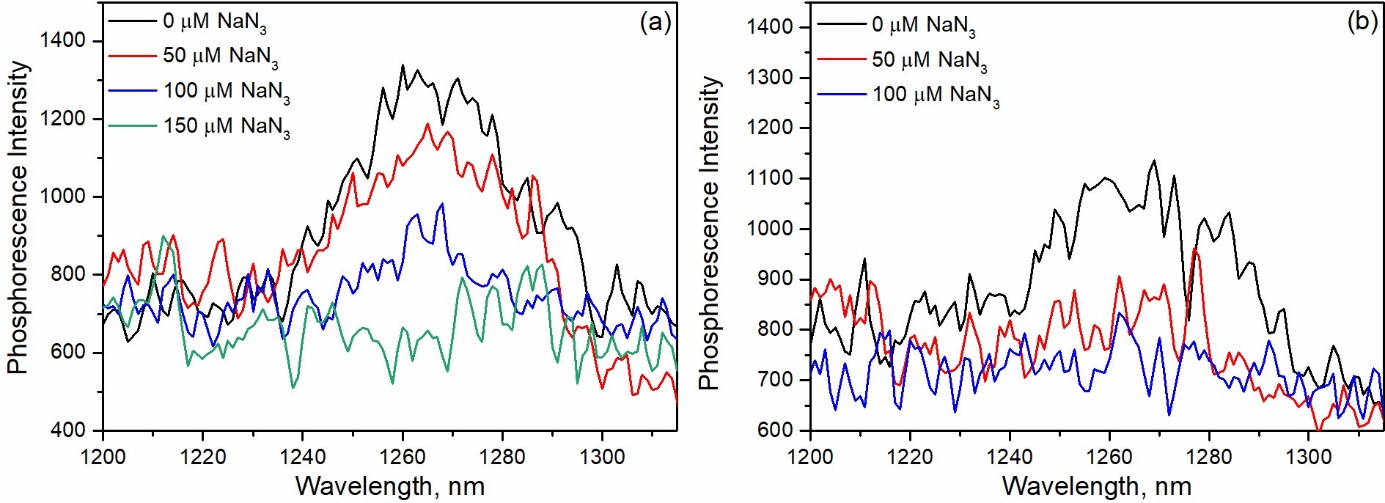


Fig. S36 Singlet oxygen phosphorescence at 1270 nm of a) 7 and b) 8 with the increase concentration of NaN_3_.
